# Multiscale Design of Dual-Gradient Metamaterials Using Gel-Mediated 3D-Printed Graphene Aerogels for Broadband Electromagnetic Absorption

**DOI:** 10.1007/s40820-025-02005-7

**Published:** 2026-01-05

**Authors:** Xiong Lv, Changfeng Li, Ge Wang, Diana Estevez, Junjie Yang, Qian Chen, Faxiang Qin

**Affiliations:** 1 Institute for Composites Science Innovation (InCSI), School of Materials Science and Engineering, Zhejiang University, Hangzhou, 310027 People’s Republic of China; 2 Zhejiang Key Laboratory of Advanced-Composites and Structures, Hangzhou, 310027 People’s Republic of China; 3 Ningbo Innovation Centre, Zhejiang University, Ningbo, 315100 People’s Republic of China

**Keywords:** Electromagnetic wave absorption, Gel-mediated porous graphene aerogel, Dual-gradient regulation, Direct ink writing

## Abstract

Three-dimensional (3D)-printed graphene aerogels hold promise for electromagnetic wave absorption (EWA) engineering due to its ultralow density, outstanding electromagnetic dissipation with the flexibility and precision of manufacturing strategies. However, their high conductivity causes severe impedance mismatch, limiting EWA performance. 3D printing requirements also constrain the dielectric properties of printable graphene inks, hindering the integration of high-performance absorbers with advanced manufacturing. This study proposes a polyacrylic acid (PAA) gel-mediated 3D porous graphene oxide (GO) aerogel multiscale regulation strategy. Precise gel content control enables dual-gradient tuning of the rheology (Benefiting direct ink writing (DIW)) and dielectric loss (Enhancing EWA) of GO/PAA composites and reduces aerogel density (6.9 mg cm^−3^ from 28.2 mg cm^−3^). Thermal reduction decomposes PAA into amorphous carbon nanoparticles anchored on reduced graphene oxide (rGO), enhancing impedance matching and absorption via synergistic 0D/2D interfacial polarization and conductive loss. The optimized rGO/PAA aerogel achieves a minimum reflection loss (RL) of −39.86 dB at 2.5 mm and an effective absorption bandwidth (EAB) of 8.36 GHz (9.64–18 GHz) at 3.2 mm. Combining DIW and this aerogel, we design a metamaterial absorber (MA) with dual material (dielectric loss) and structural gradients. This MA exhibits an ultrawide EAB of 14 GHz (4–18 GHz) with a total thickness of 7.8 mm. This work establishes a coupled design paradigm of “composition-structure-performance,” providing an engineerable solution for developing lightweight, broadband EWA materials.

**Supplementary Information:**

The online version contains supplementary material available at 10.1007/s40820-025-02005-7.

## Highlights

- The rGO/PAA aerogel achieves synergistic optimization for direct ink writing printing and construction of 0D/2D heterostructures in rGO sheets.
- Optimal reflection loss of −39.86 dB and effective absorption bandwidth (EAB) of 8.36 GHz are obtained with low density of 4.8 mg cm^−3^.
- Realization of an ultra-broadband metamaterial absorber of 14 GHz EAB at 7.8 mm thickness, across the C, X, and Ku bands.

**Supplementary Information:**

The online version contains supplementary material available at 10.1007/s40820-025-02005-7.

## Graphical abstract

**Figure:**
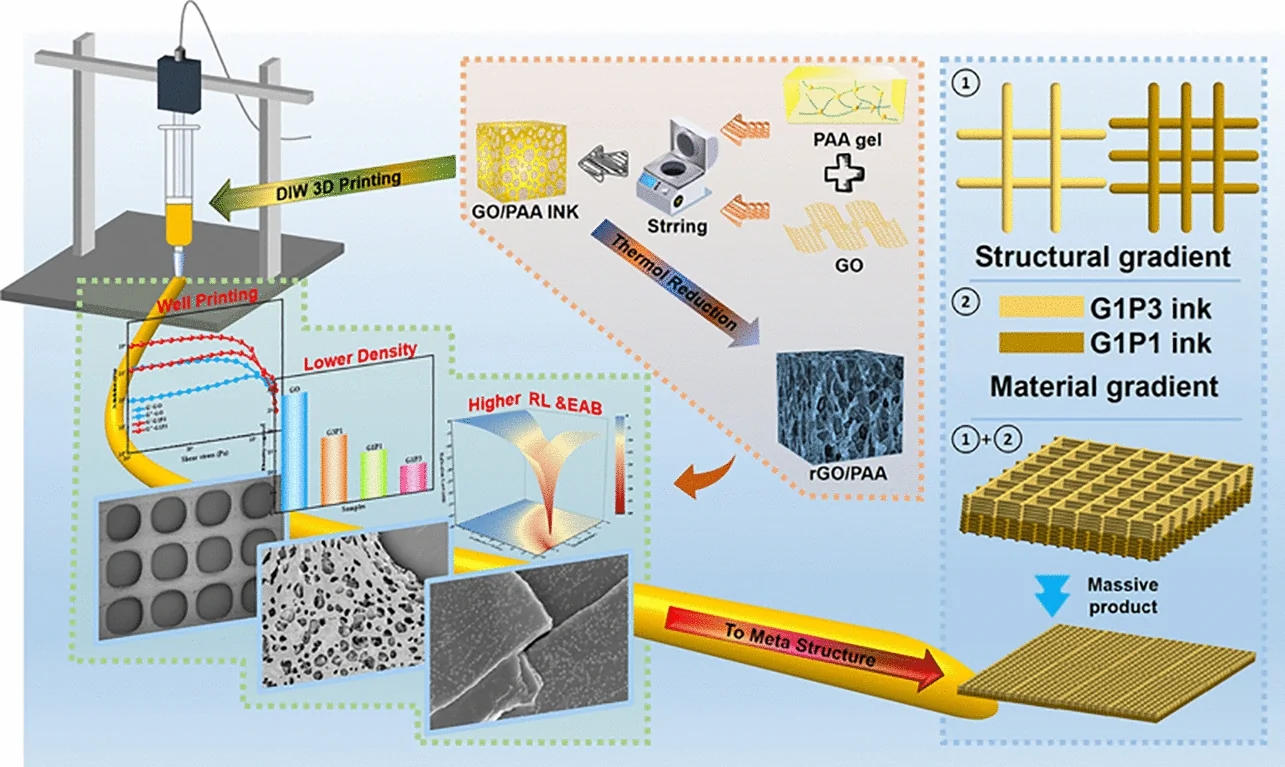

## Introduction

The widespread application of fifth-generation (5G) communication technology has intensified the demand for broadband EWA materials, which need to effectively suppress radiation pollution [1, 2] and signal interference to ensure reliable operation of electronic systems [3–5]. Traditional single-layer absorbing materials face inherent physical limitations in achieving broadband absorption due to thickness constraints governed by the quarter-wavelength principle [6–8]. Graphene aerogel [9, 10], as a porous, highly conductive, fluffy lightweight structure constructed from two-dimensional nanosheets [11, 12], facilitates multiple scattering of electromagnetic wave (EMW) within its three-dimensional network and resistive loss along conductive sheets [13–15]. These attributes position it as a high-performance absorber combining low density with strong, broadband absorption capability [16–20]. However, its excessively high intrinsic dielectric constant and dielectric loss often cause severe impedance mismatch, prompting researchers to optimize electrical properties through various modification approaches. For example: heteroatom doping (Shao et al. [21]. achieved ultralight N, S-doped graphene aerogel via pyrolysis of pyrrole and thiourea molecularly cross-linked graphene aerogel, where heteroatom-doped cell walls generated abundant dipole/defect polarization sites synergistically enhancing microwave attenuation); ceramic/graphene composite aerogels (Li et al. [22]. developed flexible SiO₂/rGO composite aerogels enabling ultra-wideband absorption with enhanced elasticity); and additional modification strategies including magnetic material/graphene [23], MXene/graphene [24], and polymer/graphene composites [25, 26]. Nevertheless, methods involving increased loading [16, 17, 27, 28] or material compositing [5, 24, 25, 29] significantly diminish the efficacy of intrinsic graphene as a two-dimensional nanomaterial while substantially increasing aerogel density and reducing stability [30], which proves detrimental for high-performance EWA. Furthermore, traditional EMW absorbers fabrication strategies predominantly focus on absorption performance while neglecting the importance of preparation processes [31–33] and production yield for practical EWA materials [34–36].

In recent years, 3D metamaterials have provided outstanding EWA solutions by synergizing intrinsic material properties with artificial topological structures [37, 38]. These architectures leverage secondary diffraction resonance and optimized impedance matching to broaden the EAB in microwave frequencies [39, 40], while their customizable geometric features simultaneously create conditions for multifunctional integrated design. Notably, the design freedom enables the creation of robust metamaterial structures that can sustain functionality under extreme mechanical and thermal conditions, paving the way for next-generation flexible absorbers applicable in harsh environments [41–44]. However, the practical fabrication of such metamaterials faces significant challenges, particularly in manufacturing complex hierarchical structures that require multilevel coordination among diverse material selections and macroscopic device configuration designs. Coordinated control of factors such as "material properties, structural topology, performance optimization and manufacturing methods" often falls short of initial objectives. In component production, traditional mold forming involves complex injection molding and demolding procedures, substantially increasing manufacturing costs while compromising product robustness [45, 46]. DIW [11, 12, 18, 38, 47, 48] within additive manufacturing technology offers a transformative platform to overcome these limitations [49, 50]. As an extrusion-based 3D printing technique, DIW demonstrates exceptional compatibility [47, 51] with various functional materials by regulating the rheological properties of precursor inks [52]. This technology enables microstructure control with micron-level precision while preserving critical material characteristics, providing an unprecedented design approach for constructing metamaterials with programmable electromagnetic responses [50]. DIW uniquely integrates design freedom, multifunctionality, and stability into printed structures [53, 54]. Furthermore, its potential for single-step processing of multi-material structures simplifies overall manufacturing time, energy consumption, cost, and waste without compromising essential material properties [55]. Thus, DIW technology offers immense potential for processing 3D metamaterials and enabling large-scale industrial production.

Herein, we demonstrate a unique graphene aerogel composite approach, combining rGO/PAA with DIW technology to customize an ultralight graphene aerogel with broadband EWA and propose an integrated material-structure dual-gradient design method for 3D metamaterial absorbers (TOC). First, we precisely constructed a series of GO/PAA composite aerogels (named as GxPy, GO: PAA = x: y) with different ratios. The introduction of high-water-content PAA gel significantly alters the pore structure of the composite aerogel, consequently inducing a gradient change in dielectric constant and corresponding modulation of the material's electromagnetic properties; second, benefiting from higher water content, the density of the composite aerogel obtained after freeze-drying is significantly reduced to one-fourth that of pure graphene oxide aerogel. Additionally, after thermal reduction, the amorphous carbon particles formed by the pyrolysis of PAA in the composite aerogel grow uniformly on the graphene sheets, forming 0D/2D interface effect and enhancing EMW. Simultaneously, we characterized the changes in the GO sheet structure, and the resulting enhancement in rheological properties enables its use as a water-based ink suitable for DIW, integrated into the high-performance absorber reduced G1P3 (rG1P3). Finally, combining the composite ink with DIW, we propose a design method for an integrated material-structure dual-gradient metastructure absorber, with simulation and testing data indicating broadband absorption potential. Through aerogel 3D stacking sequence control, 2D sheet surface modification, and metamaterial macrostructure design, the resulting MA exhibits an EAB of up to 14 GHz (4–18 GHz). Utilizing this multi-scale design method, combining ink rheological design, aerogel dielectric design, metamaterial topological structure, and DIW technology provides a unique approach for the design and production of high-performance broadband EMW absorbers.

## Experimental Section

### Materials

Expanded graphite (EG, 80 mesh, ∼50 μm) was purchased from Qingdao Tengshengda Graphite Co., Ltd. China. Potassium permanganate (KMnO_4_), hydrogen peroxide (H_2_O_2_), sulfuric acid (H_2_SO_4_) and dichloromethane were obtained from Sinopharm Chemical Reagent Co., Ltd., China. Carbopol 940 were obtained from Hangzhou Chentong Biochemical Technology Co., Ltd., China. All chemical reagents were of analytical grade and not further purified.

### Preparation of GO, PAA, GxPy ink, rGxPy aerogels and Dual-Gradient MA

#### Preparation of the GO

GO aqueous dispersion was synthesized via a modified Hummers method [56, 57]: First, 1 g of 80 mesh expanded graphite was gradually added into 80 ml concentrated H₂SO₄ under mechanical stirring (500 rpm) to form a homogeneous mixture. Then, 4 g KMnO₄ was added in batches at a constant rate. After complete dissolution, the reaction system was transferred to ambient temperature and continuously stirred at 500 rpm for 12 h, with periodic removal of residual deposits on container walls. Post-reaction, the system was re-immersed in an ice bath, while 100 mL deionized water was slowly dripped under mechanical agitation, followed by controlled addition of 50 mL 30% H₂O₂ solution. Purification was performed through gradient centrifugation (6,000–10,000 rpm, 6 min) until the supernatant reached neutral pH. Homogenization and bubble removal were achieved using a planetary homogenizer (AR-100, THINKY) at 3,000 rpm for 60 s.

#### Synthesis of GO/PAA Composite Inks

A transparent PAA gel precursor was prepared by dispersing 1 g Carbopol 940 powder in 200 mL deionized water under mechanical stirring (800 rpm), followed by 12 h hydration. The system pH was adjusted to 8 through dropwise addition of 10% NaOH solution, with subsequent 12 h stirring at ambient temperature to ensure complete neutralization [58–60]. The resultant gel was degassed using the planetary homogenizer at 2,000 rpm for 15 s. GO/PAA composite inks with gradient compositions were formulated by blending PAA gel with GO aqueous dispersion at mass ratios of 1:3, 3:5, 1:1, 5:3, 3:1, and 7:1 (labeled as G1P3, G3P5, G1P1, G5P3, G3P1, G7P1, respectively), alongside a pristine GO dispersion control. Each mixture underwent cyclic homogenization (2,000 rpm, 15 s/cycle, 3 cycles) and degassing to achieve uniformly dispersed gradient composite inks. The filler content was determined by uniformly mixing a 0.5% concentration Carbomer solution with a 2% concentration GO solution at x: y volume ratio and calculating the total mass percentage of the dispersed phase in the mixed solution.

#### DIW of GO/PAA Aerogels

The GO/PAA composite inks were loaded into 20 mL polypropylene syringes and mounted on the DIW 3D printer (Shenzhen Microcosm 3D Technology Development Co., Ltd, Microcosm-10X). Multilayer lattice structures were programmed via G-code with the following parameters: 1–5 printing layers, 20 mm s^−1^ nozzle speed, 250–1200 μm inner diameter needles, and dynamically adjusted extrusion pressure (0.1–50 kg cm^−2^) based on real-time rheological feedback. Printed constructs were immediately flash-frozen in liquid N_2_, followed by vacuum freeze-drying (-80 °C, 1 Pa) for 18 h using an freeze dryer (LABCONCO). Thermal reduction was conducted in a tube furnace under high-purity argon atmosphere with a heating rate of 5 °C min^−1^ to 400 °C (1 h holding time), simultaneously achieving GO reduction and PAA template pyrolysis.

#### Simulation, Fabrication and Testing of rGO/PAA Aerogels and Dual-Gradient Metamaterial Absorber

The radar cross section (RCS) of the rGO/PAA aerogels under realistic far-field conditions was simulated using CST Studio Suite 2021. The simulation model featured a double-layer panel measuring 150 × 150 mm^2^, comprising a 2.5-mm-thick aerogel absorption layer on top of a 0.5-mm-thick perfect electric conductor (PEC) layer. The model was oriented in the X-O-Y plane and irradiated with a plane EMW propagating along the negative Z-axis. The angular range of incidence was varied from −90° to 90°. Open boundary conditions were imposed in all directions to replicate far-field effects [61].

Based on the principle of multi-level impedance gradient matching and the theory of multi-mechanism in EWA [62–65], a material-structure dual-gradient graphene aerogel metamaterial unit cell was designed. Utilizing the CST Studio Suite 2021, the measured rGxPy dielectric constant parameters were first input into the system. The frequency domain solver was employed, with boundary conditions configured as unit cell boundaries; the model bottom was defined as a PEC. Subsequently, solving and parametric optimization were performed within the 2–18 GHz frequency range: Systematic scanning of key geometric parameters was conducted using the built-in parameter sweep and optimization module (Figs. S11–S14). The optimization objective was to minimize the reflection loss (S_11_). After selecting the optimal parameter combination, waveguide samples of varying sizes (Table S1) were fabricated using DIW technology, the post-treatment of the metamaterials used the same process as rGO/PAA; the MA after thermal reduction was impregnated with vacuum paraffin and cut to fit the waveguide dimensions. The waveguide (Agilent N5224B) testing was used to measure the S-parameters (S_11_, S_21_) of the samples, based on which the reflection loss was calculated.

### Characterization

The microstructure of the material was analyzed by field emission scanning electron microscopy and energy dispersive spectroscopy (SEM, EDS, ZEISS GEMINI 300, accelerating voltage 5 kV) and transmission electron microscopy (TEM, FEI Talos F200s). The evolution of the crystal structure was analyzed using X-ray diffraction (XRD, Rigaku SmartLab, Cu Kα radiation source, *λ* = 1.54178 Å, scan rate 5°min^–1^, 2θ = 5°−80°). Chemical composition characterization included: Fourier transform infrared spectroscopy (FT-IR, Thermo Scientific Nicolet iS20, ATR mode, resolution 4 cm⁻^1^, 32 scans), X-ray photoelectron spectroscopy (XPS, Thermo Scientific K-Alpha, Al Kα monochromatic source, binding energy calibrated against C 1*s* peak at 284.8 eV), and micro-confocal Raman spectroscopy (532 nm laser, 100 × objective, integration time 10 s, 5 accumulations) to quantitatively evaluate defect density (I_D_/I_G_). Thermogravimetric analysis (TG, TA-Q500, temperature range 50–900 °C, heating rate: 10 °C/min, balance sensitivity: 0.1 μg, furnace atmosphere: nitrogen). Electrical conductivity measurements were carried out using the four-probe technique (PROBES TECH, RTS-8). Density measurement was determined by uniformly placing the composite ink in a fixed-volume container and measuring the mass-to-volume ratio of the aerogel (Fig. S10a). Rheological properties were determined by rotational rheometer (HAAKE, RS6000, 25 °C, cone-plate diameter 20 mm, gap 0.1 mm) within a shear rate range of 0.1–1000 s⁻^1^ [11, 66]. Electromagnetic parameters were obtained by a vector network analyzer (Agilent N5224B, 2–18 GHz, coaxial method). Sample preparation followed: after vacuum impregnation with molten paraffin (80 °C, 0.1 MPa, 10 min), samples (Fig. S10c) were cut into coaxial rings with precision molds (inner diameter Φ_in_ = 3.04 mm, outer diameter Φ_out_ = 7.00 mm).

## Results and Discussion

### Synthesis and Characterization of rGO/PAA Aerogels

**Fig. 1:**
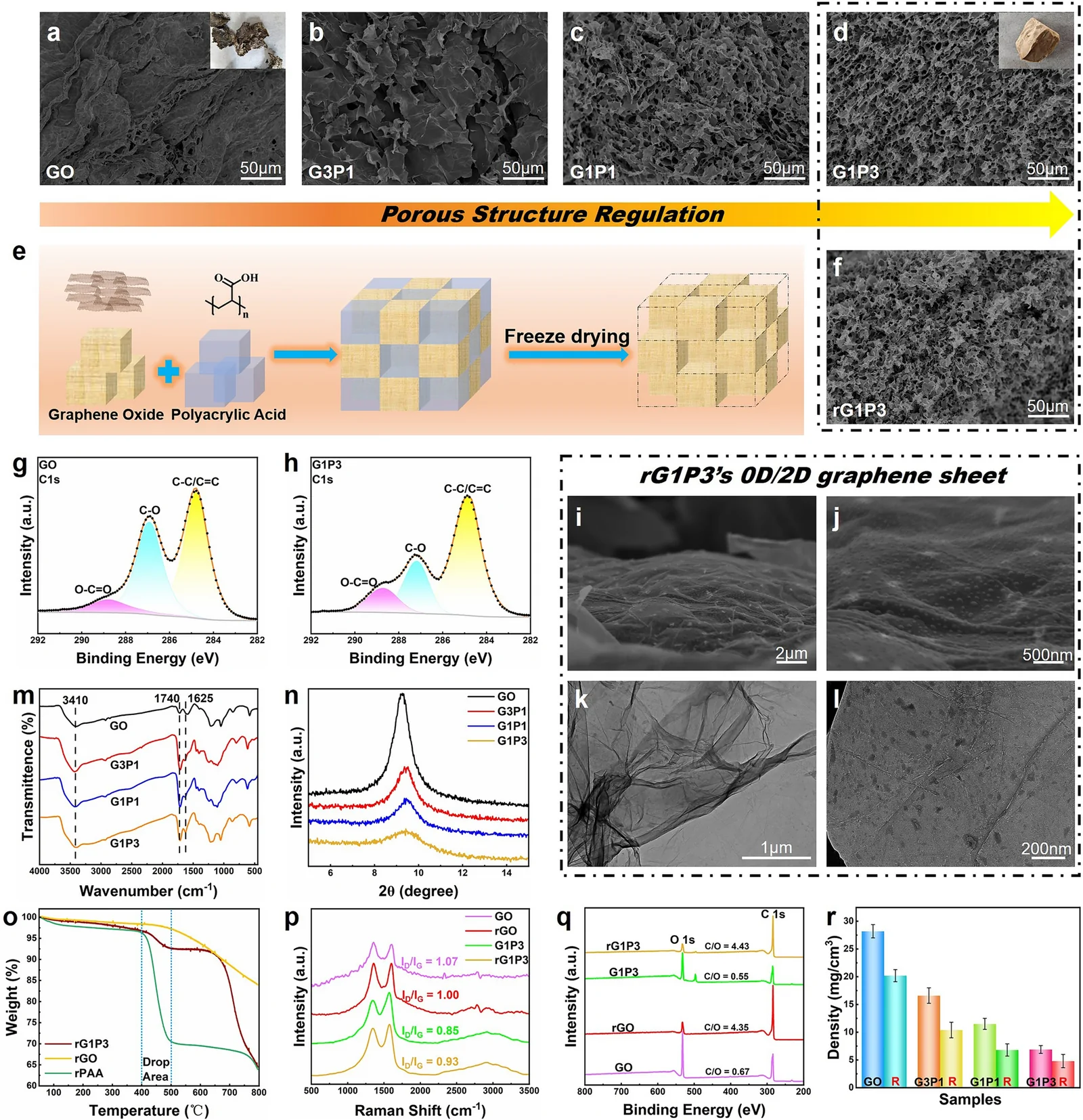
Structural evolution and compositional characterization of GO/PAA aerogels. SEM images of **a** GO, **b** G3P1, **c** G1P1, **d** G1P3. **e** Schematic illustration of structural units in composite aerogels. **f** SEM image of rG1P3. C1s XPS spectra of **g** GO aerogel, **h** G1P3 aerogel. **i, j** SEM of 0D/2D heterogeneous interface on graphene sheet layer in rG1P3. **k, l** TEM of rG1P3 nanosheet. **m** FTIR spectra of GO, G3P1, G1P1, G1P3. **n** XRD patterns of GO, G3P1, G1P1 and G1P3. **o** TGA spectra of rGO, rG1P3 and rPAA. **p** Raman spectra and **q** XPS survey spectra of GO, G1P3, rGO, and rG1P3. **r** Density of (R)GO, (R)G3P1, (R)G1P1 and (R)G1P3 aerogels, “R” represents aerogel after thermal reduction

The GxPy series aerogels were prepared by mechanically mixing GO and PAA gel in corresponding ratios (Fig. S1), followed by freeze-drying. A gradient increase in PAA content implies a reduced relative proportion of GO in the composite, significantly affecting the dispersion uniformity and stacking order of GO sheets within the three-dimensional network. The morphology of the prepared aerogels was observed via SEM (Fig. 1a–d). All GO/PAA composite aerogels exhibited a 3D porous structure. However, the pure GO aerogel primarily showed highly disordered random stacking of large GO sheets (Figs. 1a and S2). Upon the introduction of PAA, although large-sheet stacking persists in G3P1 (Fig. 1b), the porosity increases relative to pure GO, along with a noticeable reduction in sheet stacking density. As the PAA content rises further (G1P1 and G1P3), the aerogel structure becomes progressively looser and more porous, accompanied by a significantly improved spatial uniformity in GO sheet distribution (Fig. 1c, d). However, an excessively high PAA ratio dilutes and weakens the robust GO skeletal network (Fig. S3), rendering it incapable of withstanding the substantial capillary forces generated during freeze-drying, ultimately leading to structural collapse. The morphological evolution across the series clearly demonstrates the gradient improvement in GO dispersion with increasing PAA content. This improvement primarily stems from the role of PAA gel: The 3D network formed by PAA polymer chains and water acts as an effective dispersion medium, embedding and isolating GO sheets, thereby markedly reducing direct interactions and stacking tendencies between GO layers. Concurrently, the rheological properties of PAA gel (e.g., high viscosity and gel strength) not only stabilize the dispersed GO sheets but also provide the viscoelasticity required for DIW of composite inks.

XPS C1*s* spectra compared the functional groups and contents of GO and G1P3 composite aerogels (Fig. 1g, h). The intensity of the O–C = O peak (~ 288.8 eV) assigned to carboxyl/carbonyl groups in G1P3 significantly increased, indicating abundant carboxyl groups from PAA on the composite surface/interface. Combined with the alkaline environment of PAA (preventing esterification between -COOH and -OH in GO), this provides key evidence for hydrogen bonding between -COOH/-COO⁻ of PAA and oxygen-containing functional groups (e.g., -OH, -COOH) of GO, which enhances ink stability. FTIR further supports this conclusion (Fig. 1m). Key characteristic peaks include [67]: 3410 cm⁻^1^ (O–H stretching vibration from GO, PAA, and adsorbed water), 1740 cm⁻^1^ (primarily C = O stretching of carboxylic acid in PAA), and 1625 cm⁻^1^ (*sp*^2^ carbon skeleton vibration of GO). As PAA content increased, the intensities of the C = O peak at 1740 cm⁻^1^ and the broad O–H peak at 3410 cm⁻^1^ notably strengthened, consistent with successful PAA incorporation and hydrogen bonding with GO. Raman spectra (Fig. 1p) showed typical D-band (~ 1353 cm⁻^1^, defect-induced) and G-band (~ 1600 cm⁻^1^, *sp*^2^ carbon lattice vibration) for GO and composite aerogels. The I_D_/I_G_ intensity ratio reflects *sp*^3^ defect density; a higher ratio indicates more defects. The I_D_/I_G_ value of G1P3 aerogel (0.85) decreased compared to GO (1.07), indicating reduced *sp*^3^ defects, extended *sp*^2^ conjugated domains, and improved structural ordering. This structural regularization enhances the rheological behavior of the ink, enabling more uniform stress distribution during DIW extrusion, thereby improving filament uniformity and printing resolution. XRD analysis (Fig. 1n) revealed that all samples exhibited a diffraction peak near 2θ ≈ 9.6°, corresponding to the (001) plane of GO and its composites, confirming that compounding did not significantly alter the interlayer spacing of GO. However, the full width at half maximum (FWHM) of the diffraction peak increased with PAA content, indicating disruption of long-range ordered stacking along the c-axis (grain refinement or increased stacking disorder). This directly verifies that flexible PAA polymer inserts between GO layers or coats GO sheets, inhibiting tight ordered stacking of GO (i.e., suppressing stacking defects) and forming a "flexible (PAA)-rigid (GO) composite" structure, which promotes a more uniform aerogel network.

The optimized 3D network (Fig. 1e) enhances structural stability and anti-deformation capability of ink filaments during extrusion, ensuring filament morphology. Simultaneously, this series of aerogels maintained ultralow density after drying, meeting lightweight application requirements. The most intuitive change with increased PAA content was a significant lightening of the aerogel color (Fig. S3), attributed to reduced GO proportion and altered dispersion in higher-porosity structures. Density measurements (Fig. 1r) confirmed that GxPy aerogel density decreased markedly with PAA content; G1P3 density was as low as 6.9 mg cm^−3^, approximately one-fourth that of pure GO aerogel (28.2 mg cm^−3^). The density of the rGO/PAA further decreased after undergoing thermal reduction and annealing steps; the density of rG1P3 was measured to be 4.8 mg cm^−3^, due to the partial removal of oxygen-containing groups and the contraction of the carbon skeleton. Regarding electromagnetic properties, for rGO-based materials, densely stacked structures easily yield high conductivity and impedance mismatch, hindering EMW penetration. Lower porosity also limits multiple reflections/scattering of waves, impairing loss. In contrast, introducing PAA to form highly porous, uniformly dispersed GO composite structures lays the foundation for tuning dielectric properties, optimizing impedance matching, and promoting multiple electromagnetic wave scattering, demonstrating potential for improving electromagnetic performance.

The structural and compositional evolution of the GxPy composite aerogels upon thermal annealing at 400 °C is examined in Fig. 1f. The images confirm that the rG1P3 aerogel successfully retained the loose, porous 3D aerogel architecture of its precursor, with the significant color change accompanying the reduction being visually evident in Fig. S10b. More importantly, they reveal the uniform loading of numerous 0D particles (50–100 nm) onto the rGO sheets (Fig. 1i, j), forming a distinct 0D/2D heterostructure. This morphology is directly attributable to the initial ‘gel-mediated’ strategy: the homogeneous dispersion of GO within the 3D PAA gel network predetermined the spatial distribution of the components, which, upon thermal reduction, was faithfully translated into rGO framework with in situ formed amorphous carbon nanoparticles anchored uniformly. The origin of these 0D particles was identified by analyzing the pyrolyzed pure PAA powder **(**Fig. S8c–e), which forms a porous bulk amorphous carbon with a randomly stacked morphology. Therefore, the distinctive heterostructure arises from the carbonization of the pre-organized PAA gel network. This 0D/2D architecture is pivotal for tailoring dielectric properties. The carbonization of PAA at 400 °C produces amorphous carbon nanoparticles rich in defects and residual dipolar groups. While the rGO sheets provide basal conductivity, the incorporated amorphous carbon nanoparticles introduce significant dipole polarization. Crucially, the pronounced electrical conductivity contrast between the semiconducting 0D particles and the conductive 2D rGO sheets promotes intense interfacial polarization (Maxwell–Wagner–Sillars effect) at their heterojunctions, which is identified as the primary mechanism for the enhanced dielectric loss. EDS analysis (Fig. S8a, b) provides compositional evidence: Compared to pure rGO sheets, the carbon-particle-loaded rG1P3 sheets exhibit significantly higher areal carbon density, directly confirming the successful introduction and loading of PAA amorphous carbon residues. This unique 0D carbon nanoparticles/2D graphene sheets (0D/2D) heterostructure, owing to its pronounced interface effects, lays the foundation for enhanced interfacial polarization. TEM results (Fig. 1k, l) provide further microstructural evidence, confirming the formation of high-quality monolayer rGO and the attachment of amorphous carbon particles on its surface.

Thermal stability and interfacial interactions were further probed by TGA. The analysis provided critical evidence for the presence of an intimate 0D/2D interface through distinct weight loss behaviors (Fig. 1o). Below 400 °C, all samples exhibited minimal weight loss. A significant divergence occurred between 400 and 500 °C, and pure rGO experienced slight mass loss from the removal of stable oxygen groups; rPAA underwent severe decomposition of its carbonized residue; crucially, the weight loss rate of rG1P3 was intermediate between the two. This unique intermediate behavior cannot be attributed to simple physical mixing but strongly indicates the formation of a stabilizing heterointerface. We propose two interfacial effects: (1) a physical barrier effect, where rGO sheets hinder the escape of small molecules from the decomposing PAA-derived carbon; and (2) interfacial stabilization via van der Waals forces or π-π interactions. Both mechanisms originate from the close contact within the 0D/2D heterostructure. TGA performed in air further elucidated the material's oxidation resistance (Fig. S4). Although the incorporation of the PAA-derived carbon component slightly reduced the onset temperature of oxidation compared to pure rGO, the overall weight loss behavior of rG1P3 suggests a mitigating effect attributable to the rGO framework. The material exhibits excellent thermal stability at 400 °C. Additionally, Supplementary Video S1 demonstrates the hydrophobic behavior and structural fidelity of rG1P3 when immersed in water at different temperatures, confirming the material's ability to maintain its structural integrity under varying ambient conditions. The mechanisms are: (1) Physical barrier effect: rGO sheets effectively hinder the diffusion and escape of small-molecule gases (H₂, CH₄, CO, etc.) generated by the decomposition of PAA carbon residues to the surface, forcing gases to detour around or penetrate the sheet network at the heterointerface; (2) Stabilization by interfacial interactions: Potential physical adsorption (e.g., van der Waals forces) or π-π conjugation interactions between rGO and amorphous carbon particles may partially stabilize the structure of the carbon residue, making deep cracking more difficult. Both effects stem from the close contact and interfacial bonding between 0D particles and 2D sheets, a direct consequence of the formed 0D/2D heterostructure.

Raman spectroscopy (Fig. 1p) and XPS survey spectra (Fig. 1q) collectively analyzed the evolution of chemical structure and defects. The XPS results confirmed the effective thermal reduction, as evidenced by the significant increase in the C/O atomic ratio from 0.67 for GO to 4.35 for rGO, and similarly from 0.55 for G1P3 to 4.43 for rG1P3. Raman analysis revealed: (1) Thermal reduction caused the ID/IG ratio of rGO (0.93) to be significantly lower than that of pristine GO (1.07), clearly reflecting the reduction of in-plane *sp*^3^ defects and extension of *sp*^2^ conjugated domains due to thermal healing; (2) The unreduced composite G1P3 also had a lower I_D_/I_G_ ratio (0.85) than pure GO, benefiting from PAA's multiple roles: acting as a dispersant to inhibit GO aggregation and thus reduce stacking defects. Its carboxyl groups (-COOH) potentially decrease the oxygen-containing defect density in GO via partial reduction (e.g., electron transfer/hydrogen bonding); and its flexible polymer chains helping alleviate local stress concentration and lattice distortion in GO sheets; (3) The I_D_/I_G_ ratio of reduced rG1P3 (0.93) was higher than that of its precursor G1P3 (0.85). Combined with the nanoparticle loading observed by SEM (Fig. 1i, j), this increase in I_D_/I_G_ primarily stems from new *sp*^3^ defects introduced by the 50–100 nm amorphous carbon residues generated from PAA carbonization, partially offsetting the healing effect of thermal reduction on the rGO sheets themselves. XPS and XRD results further corroborated the reduction effect. The XPS analysis revealed a significant increase in the C/O atomic ratio of rGO compared to GO (Fig. S9a, b). Concurrently, the XRD pattern of rG1P3 (Fig. S9c)  exhibits  a redshift in the 2θ angle, indicating that the removal of oxygen-containing functional groups (–OH, C–O–C, C = O; accompanied by CO_2_/H_2_O release) leads to a readjustment of the interlayer spacing. This findings confirmed the restoration of the *sp*^2^ conjugated network. The increased C/O ratio is a key indicator of the reduction degree, directly correlating to significantly enhanced electrical conductivity and chemical stability, which underpins the excellent transport capability and optimized electromagnetic performance.

### Printing Performance of GO/PAA Ink

**Fig. 2:**
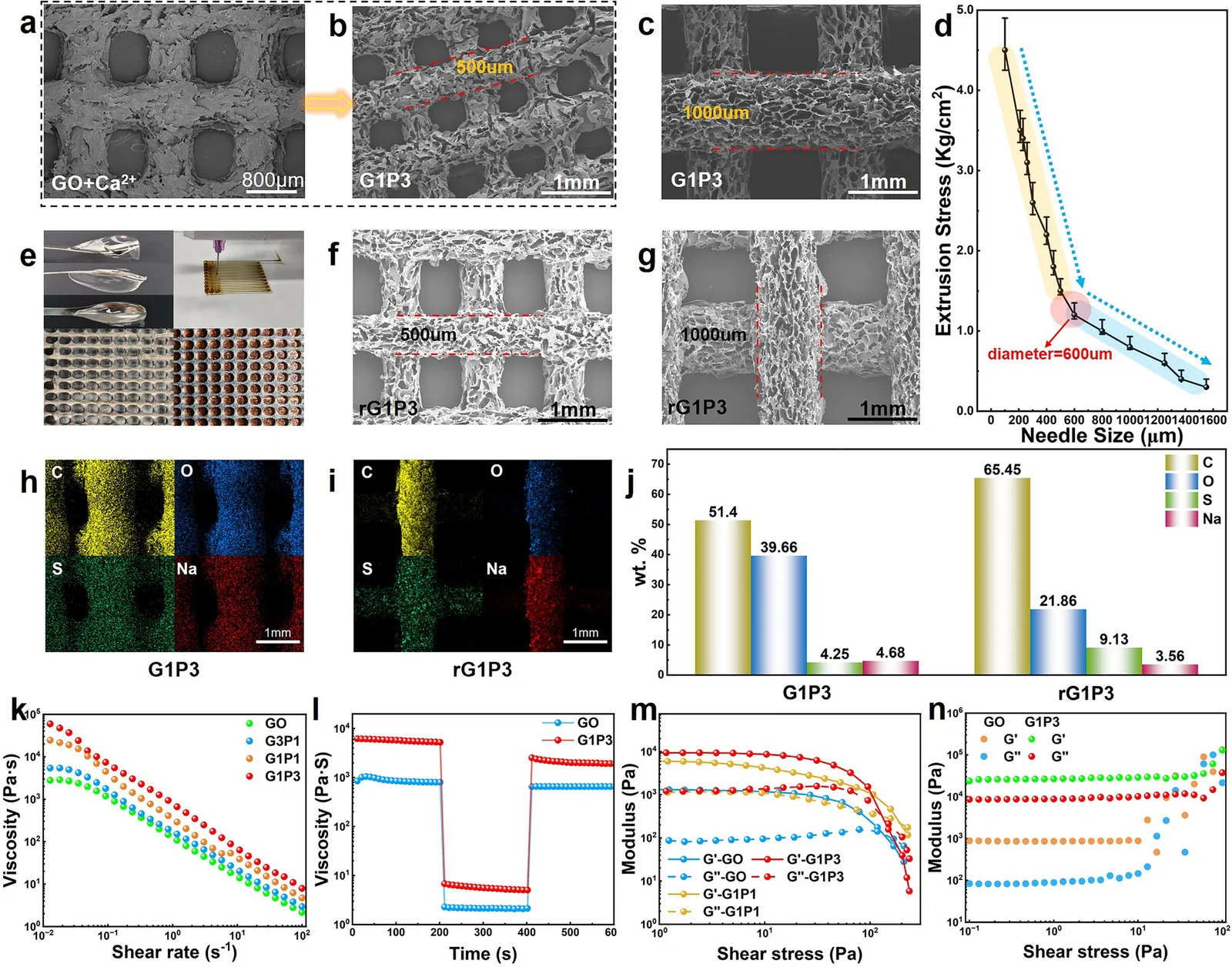
Structural evolution and multi-scale characterization of graphene frameworks. **a** SEM image of Ca^2^⁺ crosslinked framework, **b, c** SEM image of G1P3 framework, **d** The relationship between extrusion pressure and nozzle diameter. **e** Digital images of Carbopol gel matrix, 3D printing process, and frameworks before/after reduction. **f, g** SEM image of rG1P3 framework. EDS mapping of **h** G1P3 and** i** rG1P3 framework. **j** EDS test results for element weight percentages (Wt. %). **k** Ink viscosity versus shear rate. **l** Thixotropy property of inks. The dependence of storage modulus (G′) and loss modulus (G″) on **m** shear stress and **n** frequency

To integrate high-performance materials with advanced processing techniques and promote their industrialization and mass production, we describe the DIW 3D printing process of GxPy composite aerogel precursors. Figure 2 comprehensively presents the printing performance of GxPy inks (represented by G1P3) and their derived structures. To highlight the impact of composite design on printed structures, a literature-reported calcium-ion-crosslinked GO ink system (GO + 7.5 mmol mL^−1^ CaCl₂·6H₂O, the other preparation conditions are consistent) served as a control [30]. SEM images (Fig. 2a, b) reveal significant microstructural differences in the printed frameworks of the two inks: ion-crosslinked GO formed a typically dense, stacked lamellar structure, accompanied by compact laminates and faults induced by extrusion-tension forces (Fig. 2a); whereas the G1P3 ink exhibited a unique, uniformly sized, and orderly arranged open porous network structure (Fig. 2b, c). This differentiated porous structure is primarily attributed to the introduction of PAA and its regulation of in situ water sublimation pathways during freeze-drying. Furthermore, during the 3D printing process, the stress induced by nozzle extrusion influences the microstructural evolution of the printed samples, particularly the stacking behavior of GO layers, which varies with nozzle diameter. Figure 2b, c shows the microstructures of G1P3 printed using small and large nozzle diameters, while Fig. 2f, g shows the corresponding rG1P3 morphologies. Additional extrusion pressure data for different nozzle sizes are provided in Fig. S7. The results indicate that when the nozzle diameter is below 600 μm, the axial extrusion force along the printing direction dominates the alignment of graphene layers, causing them to yield and stack preferentially along the extrusion path. In contrast, when the nozzle diameter exceeds 600 μm, the extrusion pressure drops significantly (Fig. 2d), and its influence on layer arrangement becomes negligible. As a result, the printed structures exhibit a morphology closer to the intrinsic material state, and their EMW performance remain unaffected by the extrusion process.

The macroscopic printing performance was visually verified by photographs. Figure 2e demonstrates the excellent self-supporting capability of PAA gel, the real-time smooth printing process of G1P3 ink, and the status of printed frameworks before and after reduction. Figure S5 further provides G1P3-based framework structures printed at different perspectives and scales, confirming their high precision and good shape fidelity; Fig. S6 confirms the high-precision printing capability of rG1P3, demonstrating that the aerogel framework printed with a 150 μm nozzle exhibits a line width of 200 μm, further corroborating their high fidelity. After thermal annealing reduction at 400 °C, the printed framework structure densified, but its overall morphology and fine structure (Fig. 2f, g) were well preserved, demonstrating the stability of the reduction process. The EDS mapping results reveal the uniform distribution of the various elements inside the G1P3 and rG1P3 aerogels (Fig. 2h, i). Figure 2j shows the bar chart of EDS test results for element weight percentages (wt. %). It can be seen that there is a significant difference in the C/O content of the G1P3 aerogel before and after reduction. The substantial elimination of oxygen-containing functional groups led to an increase in structural defects, thus increasing the electromagnetic wave dissipation pathways.

Rheological properties are core indicators for evaluating the printability of DIW inks. Compared to pure GO, the G1P3 ink exhibited better shear-thinning behavior and a higher zero-shear viscosity (∼1 × 10^5^ Pa s, Fig. 2k), ensuring continuous extrusion during DIW. For G1P3 ink subjected to shear rate variation (0.1–100 to 0.1 s⁻^1^), rapid response and excellent stability were observed (Fig. 2l). Relative to pure GO, G1P3 ink exhibited typical non-Newtonian fluid characteristics (Fig. 2m, n): The liquid state (G'' > G') dominated at low shear stress and oscillatory frequency, while the solid state (G' > G'') dominated at high shear stress and oscillatory frequency, guaranteeing smooth extrusion and rapid solidification. Consequently, G1P3 ink enables high-precision printing. These exceptional rheological properties (high modulus, high viscosity) directly endow G1P3 ink with excellent shape retention and self-supporting capabilities, macroscopically manifested as high-precision printing of complex structures (Figs. 2e and S5) and outstanding structural stability of printed frameworks during freezing (Fig. S5e) and reduction (Figs. S5f, i, and S6).

### Electromagnetic Properties of rGxPy Series Aerogels

Based on the gel-mediated dispersion control strategy, we effectively reduced the spatial density of graphene sheets within the 3D framework, successfully achieving precise regulation of the electromagnetic parameters of aerogels. In accordance with the transmission line theory, the value of reflection loss can be determined using the following equation [68]:

1 $$\begin{array}{*{20}c} {Z_{in} = Z_{0} \sqrt {\frac{{\mu_{r} }}{{\varepsilon_{r} }}} tanh\left( {j\frac{2\pi fd}{c}\sqrt {\mu_{r} \varepsilon_{r} } } \right)} \\ \end{array}$$

2 $$\begin{array}{*{20}c} {RL\left( {dB} \right) = 20log\left| {\frac{{Z_{in} - Z_{0} }}{{Z_{in} + Z_{0} }}} \right|} \\ \end{array}$$

where *Z*_*in*_ is the input impedance, *Z*_*0*_ is the free-space impedance constant (377 Ω), *f* is the frequency, *d* is the thickness of the material, and *c* is the speed of light in vacuum. *RL* is measured in dB, and a value of less than −10 dB indicates an effective absorption rate of more than 90% for electromagnetic waves.

**Fig. 3:**
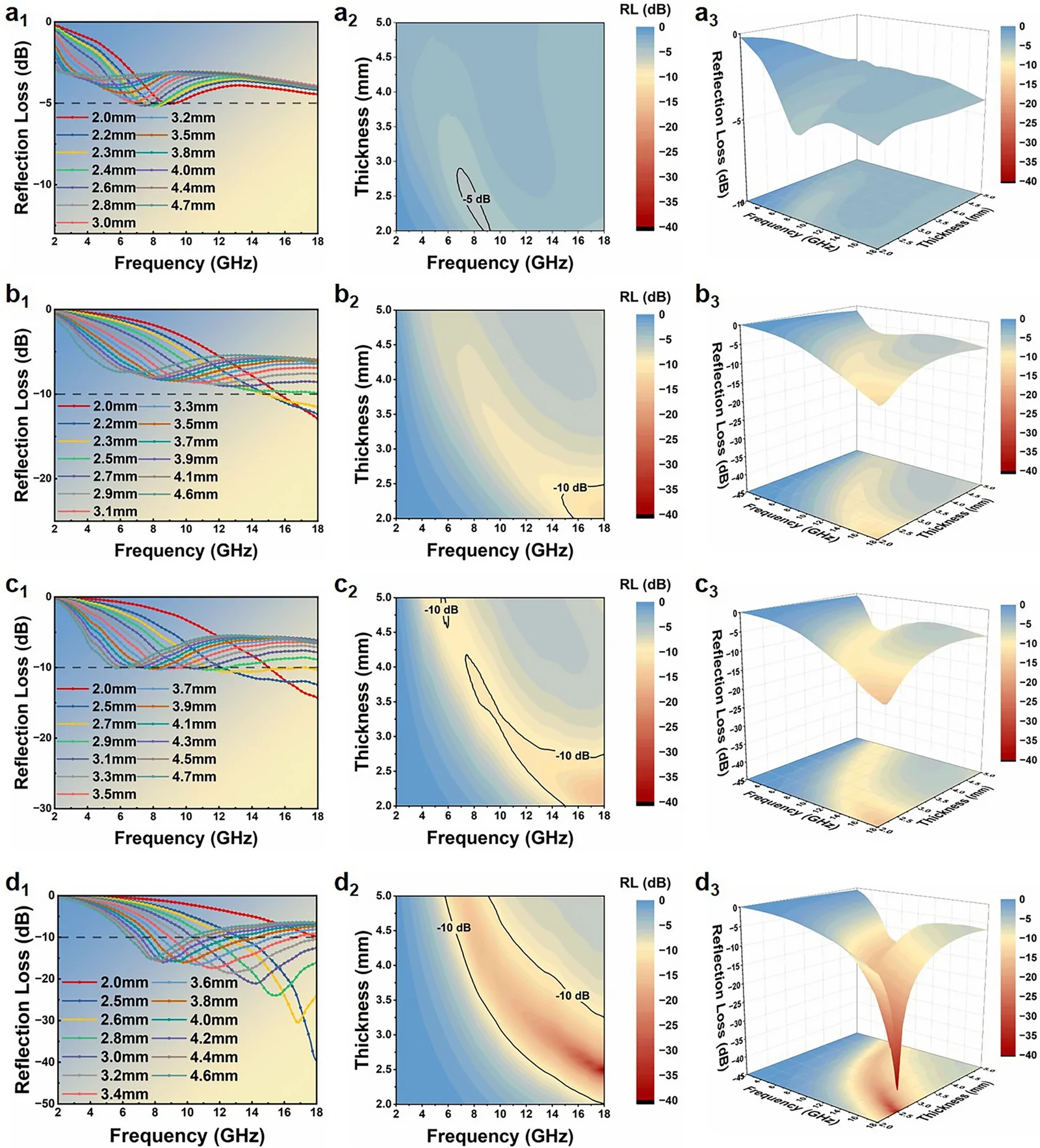
Multimodal RL analysis of graphene-based composites. **a-d** Integrated thickness-projection maps, 2D RL curves, and 3D RL plots for **a** rGO, **b** rG1P1, **c** rG3P5, and **d** rG1P3 aerogels

The EWA performance of composite aerogels was investigated by tuning composition ratios and reduction temperatures, with results shown in Fig. 3a–d. Without PAA gel, rGO exhibited RL values above −10 dB across the entire 2–18 GHz band, indicating ineffective EWA. With increasing PAA content, the EWA performance of composite aerogels significantly improved. At a GO:PAA ratio of 1:3 (rG1P3), the composite aerogel achieved the lowest RL value of -39.86 dB at 3.2 mm thickness, along with a broad EAB of 8.36 GHz at 2.5 mm thickness, covering almost the entire X (8–12 GHz) and Ku-band (12–18 GHz). Notably, the rG1P3 sample exhibited the lowest dielectric loss tangent in the aerogel series, suggesting that its broadband absorption relies not only on conductive loss but also on the constructed 3D network structure and 0D/2D interfacial polarization effects.

**Fig. 4:**
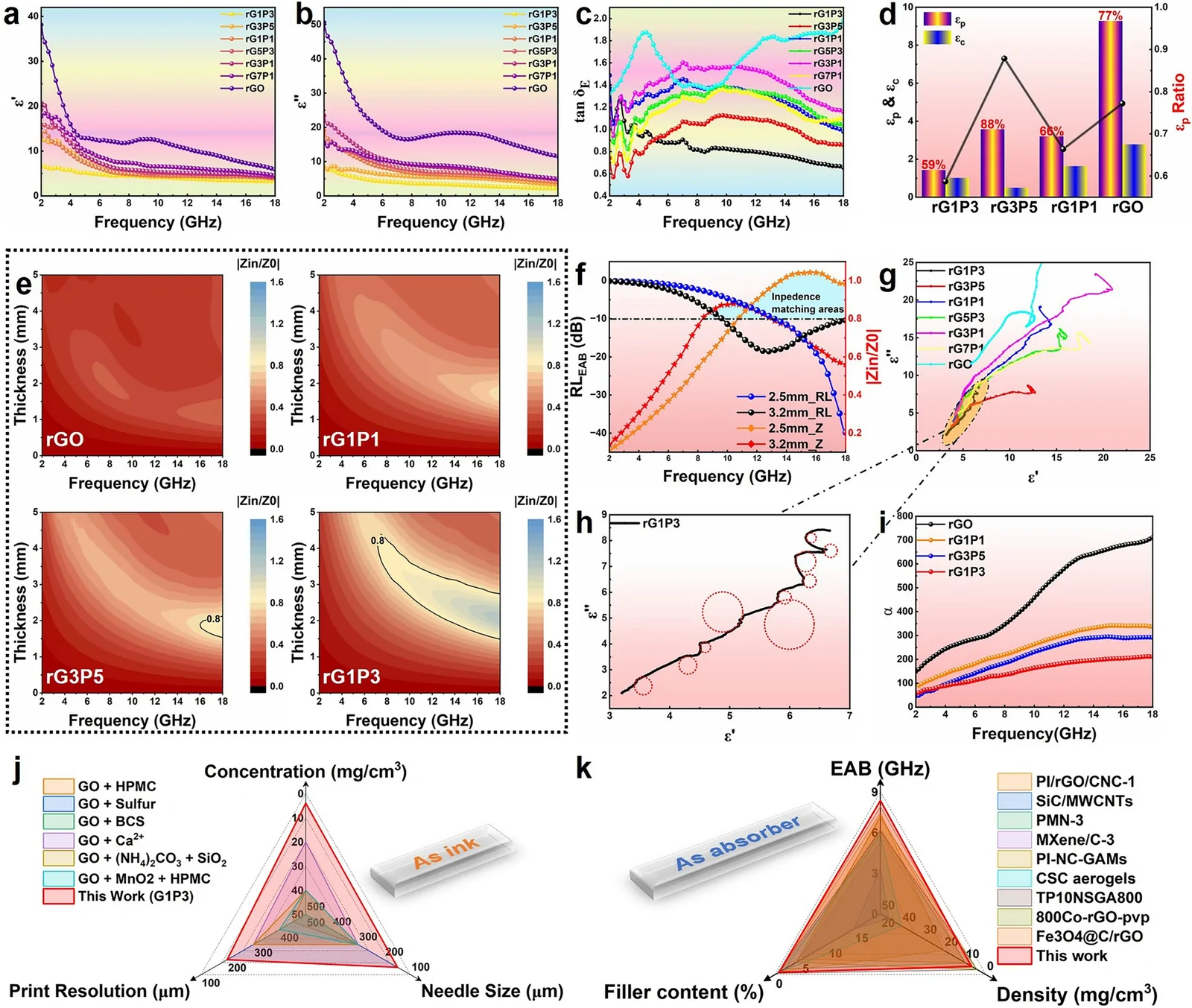
**a** Real part, **b** Imaginary part, **c** Dielectric loss of rGxPy series aerogel. **d** Conductive and polarization loss for the samples. **e** Impedance matching mappings of rGO, rG1P1, rG3P5, and rG1P3. **f** Corresponding impedance matching and RL values of rG1P3 at 2.5 mm and 3.2 mm thicknesses. **g, h** Cole–Cole plots of rGxPy series aerogel. **i** Attenuation constants of rGO, rG1P1, rG3P5, and rG1P3. **j** Comparison of the present work with those reported regarding ink. **k** Comparison of EMA performances for related 3D porous aerogel based absorbers compared with this work

To elucidate the EWA mechanism of the rG1P3 aerogel, Fig. 4a–c systematically displays the frequency dependence of the real part (*ε*′), imaginary part (*ε*′′) of complex permittivity, and dielectric loss tangent (tan *δ*ₑ = *ε*′′/*ε*′) for the rGxPy series samples in the 2–18 GHz frequency band. All samples exhibited typical dielectric dispersion behavior, where ε′ and ε′′ values monotonically decreased with increasing frequency, originating from the lagged response of polarization relaxation processes to high-frequency electric fields [69, 70]. Notably, dielectric properties could be directionally tuned by adjusting the mass ratio of GO to PAA: As PAA content increased (rG3P1 → rG1P1 → rG1P3), the amplitudes of *ε*′ and *ε*′′ decreased significantly (Fig. 4a, b). Regarding loss mechanisms, pure rGO exhibited an abnormally high dielectric loss tangent (tan *δ*ₑ) with a sharp resonance peak at ~ 4 GHz (Fig. 4c**)**, primarily attributed to strong interfacial polarization and multiple scattering effects induced by its densely stacked structure [71–73]. After introducing PAA, the tan *δ*ₑ curves of composite aerogels developed broadened hump-like profiles (first rising then falling), with peak intensity systematically weakening as PAA content increased.

This pattern of "higher PAA content leading to lower dielectric loss" strongly validates the core advantage of the gel strategy: Firstly, the 3D network formed by the carbonized residue of the PAA gel effectively inhibits graphene sheet stacking, reducing localized polarization centers; secondly, the 0D/2D heterointerface formed between PAA-derived amorphous carbon particles and graphene sheets optimizes charge relaxation behavior and attenuates resonance intensity. These two factors synergistically enable continuous and precise regulation of dielectric properties. To further confirm the attenuation mechanism, the polarization (ε_*p*_′′) and conduction (ε_*c*_′′) contributions to the dielectric loss have been calculated based on the Debye theory [74] according to Eq. (3): ″

3 $$\begin{array}{*{20}c} {{\upvarepsilon }^{\prime \prime } \left( {\upomega } \right) = {\upvarepsilon }_{p}^{\prime \prime } + {\upvarepsilon }_{c}^{\prime \prime } = \left( {{\upvarepsilon }_{s} - {\upvarepsilon }_{\infty } } \right)\frac{\omega \tau }{{1 + \omega^{2} \tau^{2} }} + \frac{\sigma }{{\varepsilon_{0} \omega }}} \\ \end{array}$$

where *σ* is the electrical conductivity, *ω* is the angular frequency, ε_0_ is the vacuum permittivity, ε_*s*_ is the static permittivity, ε_∞_ is the permittivity at the high-frequency limit, and *τ* is the relaxation time. As shown in Fig. 4d, the relative contributions of polarization loss (ε_*p*_'') and conduction loss (ε_*c*_'') reveal that rG1P3 exhibits the most balanced combination of these two loss mechanisms among the series. This balance is further corroborated by electrical conductivity measurements (Fig. S9d), which show a controlled decrease from 0.08 S m^−1^ for pristine rGO to 0.01 S m^−1^ for rG1P3. Such a reduction in conductivity, realized through the incorporation of amorphous carbon particles, helps mitigate excessive eddy current effects and improves impedance matching. Ultimately, this optimized performance stems from the unique porous architecture and effective heterointerface engineering between the amorphous carbon particles and graphene sheets in rG1P3.The impedance of aerogels with different components was calculated and analyzed. Impedance (Z = Z_in_/Z_0_) is a key parameter for evaluating microwave absorption, where Z_in_ is the input impedance and Z_0_ is the free-space impedance constant (377 Ω). When |Z| approaches 1, EMW penetrates the absorber more easily instead of reflecting at its surface. Thus, optimal impedance matching is a prerequisite for efficient microwave absorption. Figure 4e shows 2D impedance matching plots for gradient rGO/PAA aerogels. The color mapping scale reveals that rGO and rG1P1 aerogels exhibit no impedance matching regions (|Z| ≈ 1) within 0–5 mm thickness and 2–18 GHz frequency range, indicating poor EMW penetration despite their high permittivity. As PAA content increased, rG3P5 and rG1P3 aerogels demonstrated significant impedance matching trends. Specifically, rG1P3 achieved broadband impedance matching within 8–18 GHz. The mismatch at lower frequencies (2–8 GHz) can be attributed to the material's intrinsic dielectric behavior and the practical thickness being insufficient for optimal λ/4 matching at longer wavelength. Further analysis confirmed that at the thicknesses corresponding to its strongest absorption peak and broadest EAB, the impedance matching curve (Fig. 4f) approached Z_in_/Z_0_ ≈ 1, verifying the critical role of impedance matching in enhancing EWA.

The Cole–Cole semicircle model based on Debye relaxation theory was used to interpret the dielectric loss mechanisms. Multiple semicircles in the Cole–Cole curve (Fig. 4g, h) indicate diverse polarization relaxation processes in rG1P3 aerogel under EMW, including interfacial polarization and dipole polarization. Specifically, charge accumulation at the 0D/2D heterointerface between rGO sheets induces interfacial polarization. Meanwhile, multilevel pores, localized defects, and functional groups within the 3D rGO aerogel form polarization centers, triggering dipole polarization. These high polarization losses significantly enhance the dielectric loss capability of rG1P3 aerogel, thereby improving its EWA performance. The attenuation constant (*α*) is a key parameter for evaluating EWA capability; higher *α* values indicate stronger EMW attenuation. Figure 4i shows α variations for four samples across 2–18 GHz, with attenuation capability ordered as: rGO > rG1P1 > rG3P5 > rG1P3. While rGO exhibits the strongest attenuation due to its high conductivity and dielectric loss, excessive dielectric parameters cause impedance mismatch, leading to EMW reflection/transmission and ultimately weakening EWA performance. Thus, balancing the α and Z_in_/Z_0_ is crucial for superior EWA performance. Figure 4j, k compares the results of this study with recently reported DIW ink and aerogel materials; supplementary Tables S2 and S3 provide reference details. Benefiting from its ultralight porous structure and heterointerface design, rG1P3 demonstrates significant advantages in DIW parameter, EAB, filler content, and density, outperforming state-of-the-art microwave absorbers.

**Fig. 5:**
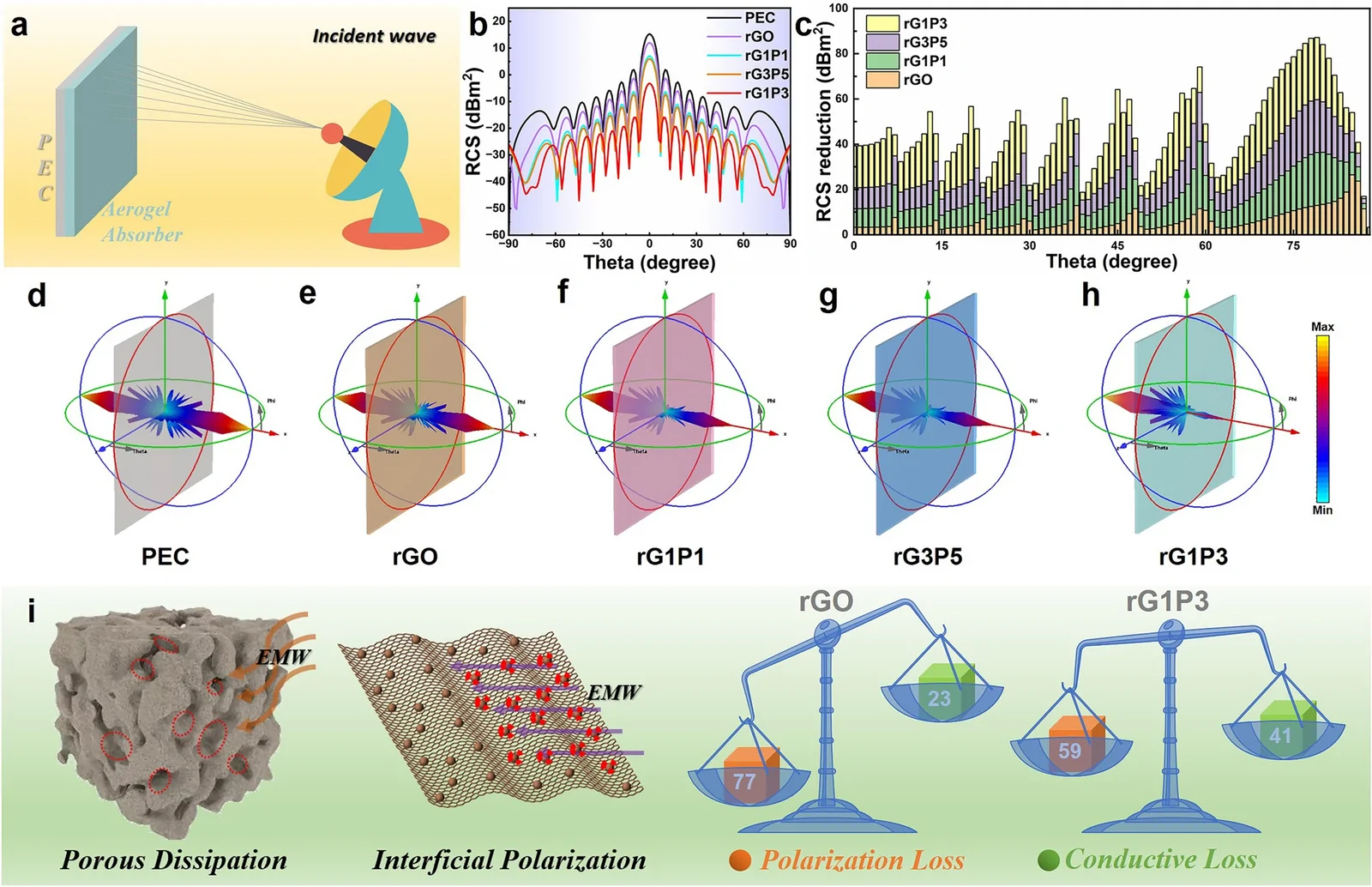
**a** Schematic diagram of radar stealth material. **b** RCS values of different aerogels. **c** RCS attenuation degree of different aerogels relative to PEC plates. **d-h** 3D images of RCS attenuation of different aerogels. **i** Schematic illustration of two loss mechanisms in rG1P3 aerogel

To systematically evaluate the far-field EWA efficacy of rGxPy composite aerogels, quantitative analysis was performed based on a RCS simulation model (Fig. 5a). Results revealed that all PEC systems coated with rGxPy aerogels exhibited significant suppression of electromagnetic scattering (Fig. 5d–h), directly confirming the material’s efficient dissipation capability for incident electromagnetic energy. Among these, the rG1P3 coating demonstrated EMW absorption performance highly synergistic with its optimal dielectric loss characteristics, its RCS distribution curve indicated stably suppressed scattering intensity below -10 dB m^2^ across 90% of detection angles (Fig. 5b). Further differential RCS analysis (ΔRCS = RCS_coating_—RCS_PEC_) more intuitively revealed that rG1P3 generated scattering reduction values over a broad incident angle (Fig. 5c). These exceptional attributes—broad angular domain coverage and strong attenuation—highlight its engineering potential. To highlight the advantages of the rGxPy aerogel system in EWA applications, the unique merits of the material design are illustrated schematically (Fig. 5i): The 3D porous structure enhances attenuation by increasing multiple internal reflections of EMW; moreover, 0D amorphous carbon particles attached to 2D graphene sheets form 0D/2D heterointerfaces, enhancing in-plane conduction loss and achieved a balance between overall conductive loss and polarization loss. This synergistic mechanism collectively promotes high-performance EWA.

### Designation of Structure-Material Dual-Gradient Aerogel MA

**Fig. 6:**
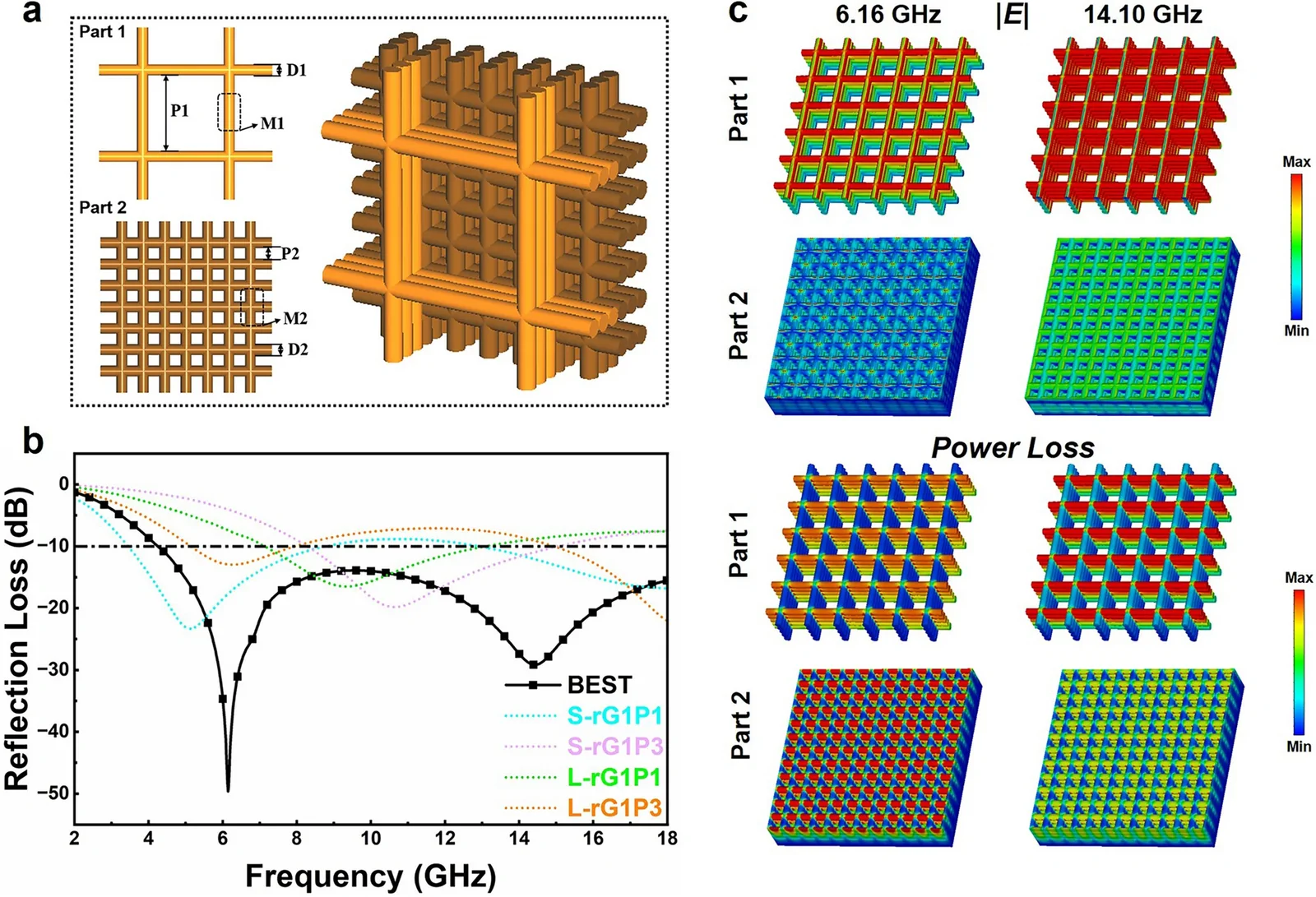
**a** Schematic diagram of metamaterial structural parameters. **b** Reflection loss obtained by simulation optimization, the black curve represents the simulation results of reflection loss for the optimal material-structure parameters. The four colored curves correspond to other types of material-structure parameter combinations (S: 1.2 mm aperture, L: 2.4 mm aperture; other parameters are consistent with the black curve). **c** Electric field distribution and energy loss density distribution of metamaterial building blocks at 6.16 GHz and 14.10 GHz

The developed rGxPy aerogel series exhibits excellent electromagnetic properties, yet planar absorbers face fundamental challenges in further enhancing broadband absorption capabilities due to the Planck–Rozanov limit [75]. To overcome this theoretical constraint and maximize the electromagnetic response characteristics of the gradient dielectric aerogel system (rG1P1/rG1P3), we constructed a novel metamaterial absorber using DIW technology through a structure-material dual-gradient design strategy. As shown in Fig. 6a, the design adopts a bilayer woodpile architecture. Structurally, the top matching layer features a large-pore loose framework that guides EMW coupling via artificially constructed hierarchical pores, while the bottom absorption layer is designed as a small-pore dense grid to enhance electromagnetic energy dissipation through high filling density. Crucially, this precise control over the 3D structural parameters (pore size P, wire diameter D) is engineered to synergize with the intrinsic 0D/2D heterointerface characteristics of the aerogels. This graded pore structure promotes multiple reflections and resonant cancelation of EMW, significantly optimizing impedance matching characteristics; Material-wise, low-dielectric-loss rG1P3 aerogel is applied to the matching layer for efficient wave impedance regulation, whereas high-dielectric-loss rG1P1 aerogel is integrated into the absorption layer to strengthen loss mechanisms, achieving cross-scale synergy between intrinsic material properties and structural functions.

Through finite-element electromagnetic simulations optimizing metamaterial parameters (Fig. 6b), the comparison between the black curve and the colored curves preliminarily validates the rationality of our design strategy (large-aperture matching layer + rG1P3 and small-aperture loss layer + rG1P1). Furthermore, parameter scanning results indicate that (Fig. S11a–f) increasing the matching layer count (N) activates low-frequency absorption peaks and induces secondary resonances (resonant peaks at 6.16 GHz and 14.10 GHz), significantly broadening the EAB. However, excessive layers (e.g., N > 5 + 5) narrow the bandwidth due to impedance mismatch. Increasing wire diameter (D) or pore size (P) causes absorption peak intensity to first strengthen then weaken nonlinearly, reaching optimal loss depth at D = 1 mm, P₁ = 2.4 mm, P₂ = 1.2 mm. This non-monotonic behavior underscores the critical match between the 3D structural parameters and the 0D/2D heterointerface-dominated loss mechanisms: An optimal structure provides the ideal spatial configuration to fully excite and utilize the interfacial polarization and conduction loss without causing detrimental impedance mismatch. The optimized configuration achieves > 90% absorption rate (covering 13 GHz EAB) at 0–45° incidence angle and maintains > 80% absorption (12 GHz EAB) within 0–60° (Fig. S11f). This performance originates from the waveguiding effect of columnar matching layers guiding EMW penetration and the high-efficiency loss of dense absorption layers. After conducting parameter decoupling optimization with higher sensitivity (Figs. S12 and S13), the final configuration was determined: layer count N = 5 + 5, wire diameters D₁ = D₂ = 1 mm, pore sizes P₁ = 2.4 mm, P₂ = 1.2 mm, with material composition rG1P3 for matching layer and rG1P1 for absorption layer. This design achieves near-perfect simulated EWA across 4–18 GHz.

To elucidate EWA mechanisms, we further simulated surface current and power loss distributions in the metamaterial unit (Fig. 6c). At the first absorption peak (6.16 GHz), electric field energy decays progressively from the top matching layer to the absorption layer depth, with current density minimized at the bottom small-pore framework. Power loss concentrates in the top and intermediate transition zones, confirming the progressive energy attenuation guided by structural gradients. At the high-frequency peak (14.10 GHz), surface currents strongly localize within the large-pore framework of the matching layer, with peak power loss occurring at the top structure, indicating that strong conductive loss from rG1P3 and multiple scattering from the porous framework jointly achieve high-frequency near-field absorption. This spatial distribution of loss directly visualizes how the macro-scale 3D structure directs EM energy to the micro-scale 0D/2D heterointerfaces within the aerogel walls, where it is ultimately dissipated. Meanwhile, Fig. S15 further shows |E| and Power Loss at the frequencies of 2, 10, and 18 GHz, which reflects the same trend. Notably, the electric field and power loss distributions highly overlap, revealing dielectric loss dominance at this frequency. In conjunction with the high-frequency EWA performance under the planar absorber, the high-frequency performance of the metamaterial is thereby explained.

**Fig. 7:**
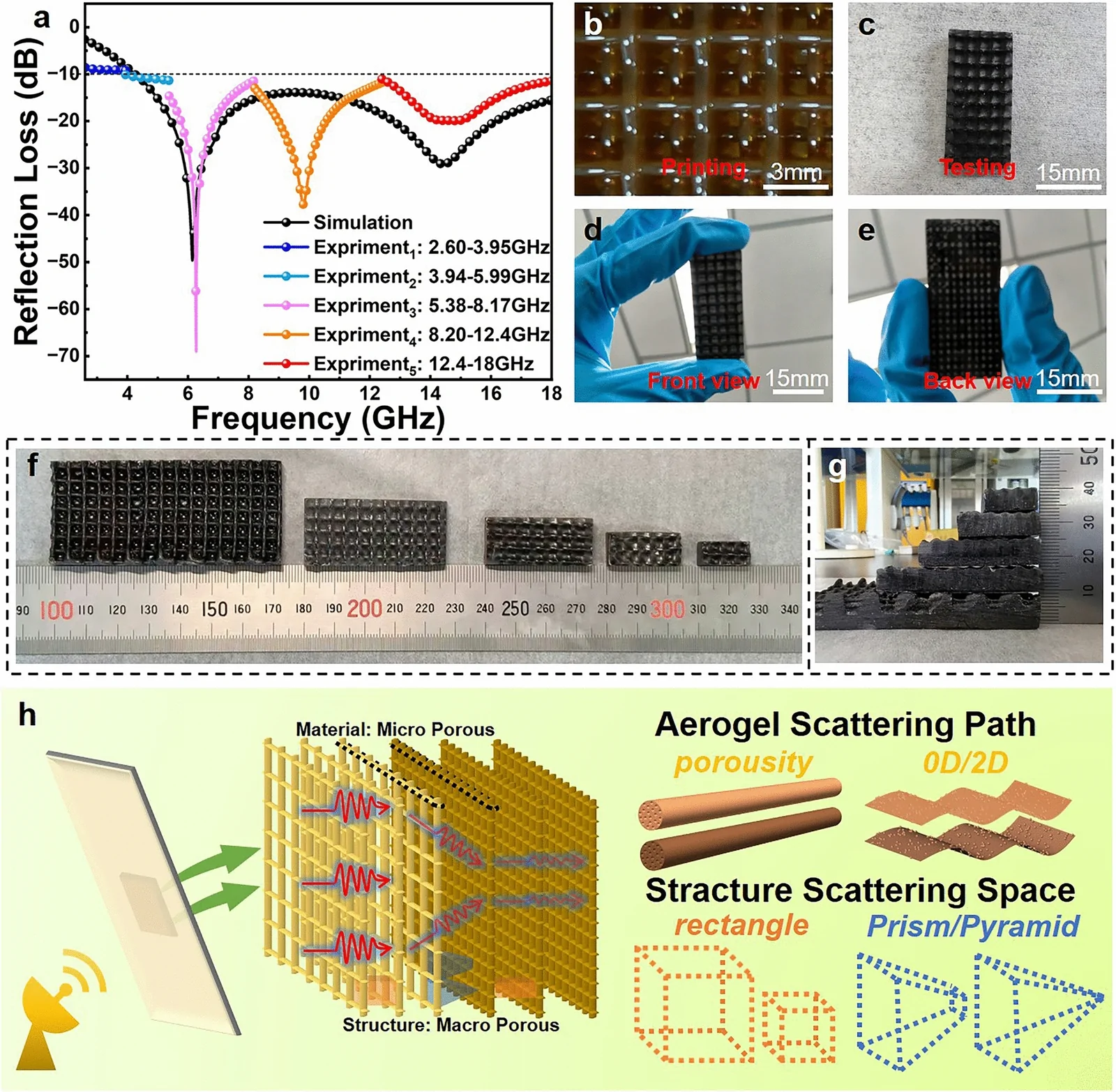
**a** Comparison of the experimental reflectivity and simulated reflectivity of the metamaterial absorber. **b** process of G1P3 ink’s DIW. **c** Overview, **d** front and **e** back view of metamaterial absorber waveguide testing samples. **f** and **g** are comparisons of the sizes of different waveguide samples, and **h** is a schematic diagram of the EWA mechanism in the material-structure integrated design

To validate the practical EWA performance of our designed metamaterial, we fabricated it into waveguide-test-compatible dimensions **(**Fig. 7f, g) and conducted tests across different frequency bands. The waveguide method was selected for its well-defined EM environment, which minimizes edge diffraction and alignment uncertainties compared to free-space methods [76, 77], thereby ensuring higher accuracy in characterizing the intrinsic absorption properties of our gradient metamaterial. Ultimately, benefiting from comprehensive optimizations in the 3D printing process and exceptionally high printing fidelity (Figs. 7b–e and S5), the absorber’s experimental reflection curves closely align with simulations (Fig. 7a), achieving ultra-broadband EWA from 4–18 GHz. The EAB fully covers C, X, and Ku bands. Minor deviations in the curves are attributable to slight discrepancies between simulation modeling and actual fabrication with finite element model structure. The unexpected resonance peak observed around 9.8 GHz in the measured reflection spectrum originates from the coupling effects of multiple factors: The actual dimensions of the sample, processed through interlayer deposition caused by gravity factors during the DIW process, become smaller than the modeled size (10 mm), and it had a measured thickness of 7.8 mm. Against the backdrop of dielectric constant dispersion, this reduction causes the dimensions to approach the effective half-wavelength (λ/2 = 7.5 mm), thereby inducing Fabry–Pérot resonance [78]. This mechanism was confirmed through additional parameter scan simulations (Fig. S14), where reducing the wire diameter to 0.7–0.8 times its original value (corresponding to a sample thickness of 7–8 mm) consistently shifted the lower absorption peak to the 9.5–10 GHz range, validating the experimental observation. Concurrently, surface roughness introduced by DIW creates minute parasitic air gaps. These gaps distort the near-field distribution through localized scattering and impedance mismatch. Furthermore, the combined effects of the dispersive characteristics of dielectric loss in the multi-layer composite material and non-uniform interlayer coupling further perturb the energy dissipation pathways. Ultimately, these factors manifest as the observed resonance peak and its positional shift in the simulated results, representing a comprehensive simulation deviation.

As shown in the schematic diagram (Fig. 7h), the dynamic complementarity between the waveguiding regulation of the matching layer (rG1P3 + large pores) and the strong attenuation of the absorption layer (rG1P1 + small pores) embodies this multi-scale design philosophy, which is fundamentally enabled by the precise match between the 3D network parameters and the 0D/2D heterointerface properties, ultimately endowing the metamaterial with near-ideal EMW characteristics featuring broadband and wide-angle performance. This work provides an innovative solution for breaking traditional absorber bandwidth limits through cross-scale coupling of material properties and spatial structures, advancing high-performance electromagnetic stealth materials toward engineering applications.

## Conclusions

We propose an integrated DIW-based fabrication strategy for synthesizing high-performance graphene aerogels and further enhance their EWA performance through metamaterial structures, achieving integrated structural–functional design. By introducing modulated PAA gel as a support framework into ultra-low-concentration GO dispersion, we realized synergistic optimization of rheological properties, electromagnetic performance and density, while thoroughly elucidating PAA’s mechanism and its contribution to high-performance EWA. First, we revealed that PAA introduction significantly enhances the dispersion of graphene nanosheets within the 3D structure, facilitating their transformation from a relatively compact/disordered state to a uniform, fluffy, and porous architecture. This transition improves the printability of composite inks and substantially reduces aerogel density; second, studies demonstrate that PAA incorporation modifies graphene layers by partially disrupting their integrity and constructing amorphous carbon (0D)-graphene nanosheet (2D) heterointerfaces, effectively regulating the aerogel’s dielectric properties; and third, electromagnetic tests confirm the composite aerogel’s exceptional EWA capabilities. Specifically, the rG1P3 aerogel achieves a EAB of 8.36 GHz at 3.2 mm thickness and RLmin of –39.86 dB (t = 2.5 mm). Finally, using optimized G1P3 composite ink combined with the material-structure dual-gradient design concept, we designed and fabricated a dual-gradient aerogel metamaterial absorber, enabling effective EWA across 4–18 GHz. Crucially, these outstanding performances are attained without complex multi-material compositions or tedious preparation processes, representing an effective fusion of advanced material-structure design and advanced manufacturing (DIW). This design philosophy provides valuable insights for developing high-performance broadband EWAs and demonstrates broad application prospects. More importantly, it establishes a versatile platform for multifunctional material systems, potentially integrating wave absorption with other engineered properties such as thermal insulation or sensing capabilities, far beyond the scope of traditional single-function absorbers.

## Supplementary Information

Below is the link to the electronic supplementary material.

Supplementary file1 (DOCX 20490 kb)

Supplementary file2 (PPTX 11859 kb)
