# Supplementary material for: Multiscale Design of Dual-Gradient Metamaterials Using Gel-Mediated 3D-Printed Graphene Aerogels for Broadband Electromagnetic Absorption
Source: Nanomicro Lett. 2026 Jan 5;18:162. doi: 10.1007/s40820-025-02005-7 (PMC12765778; doi:10.1007/s40820-025-02005-7)
Supplement: Supplementary file 1 — Supplementary file1 (DOCX 20490 kb) [file 40820_2025_2005_MOESM1_ESM.docx]

Supporting Information for

**Multiscale Design of Dual-Gradient Metamaterials Using Gel-Mediated 3D Printed Graphene Aerogels for Broadband Electromagnetic Absorption**

Xiong Lv^1^, Changfeng Li^1^, Ge Wang^1^, Diana Estevez^3^, Junjie Yang^1^, Qian Chen^1^ and Faxiang Qin^1, 2, 3^ *

^1^ Institute for Composites Science Innovation (InCSI), School of Materials Science and Engineering, Zhejiang University, Hangzhou 310028, P. R. China

^2^ Zhejiang Key Laboratory of Advanced-Composites and Structures, Hangzhou 310027, P. R. China

^3^ Ningbo Innovation Centre, Zhejiang University, Ningbo 315100, P. R. China

*Corresponding author. E-mail: [faxiangqin@zju.edu.cn](mailto:faxiangqin@zju.edu.cn) (Faxiang Qin)

**S1** The photos of the PAA gel **(Fig. S1a)** and the GO solution with a concentration of 1.6% **( Fig. S1b)** are shown. After being mixed in a certain proportion **(Fig. S1c, d)**, the composite ink has undergone a color change and also exhibited the properties of a colloid. The two have good compatibility.


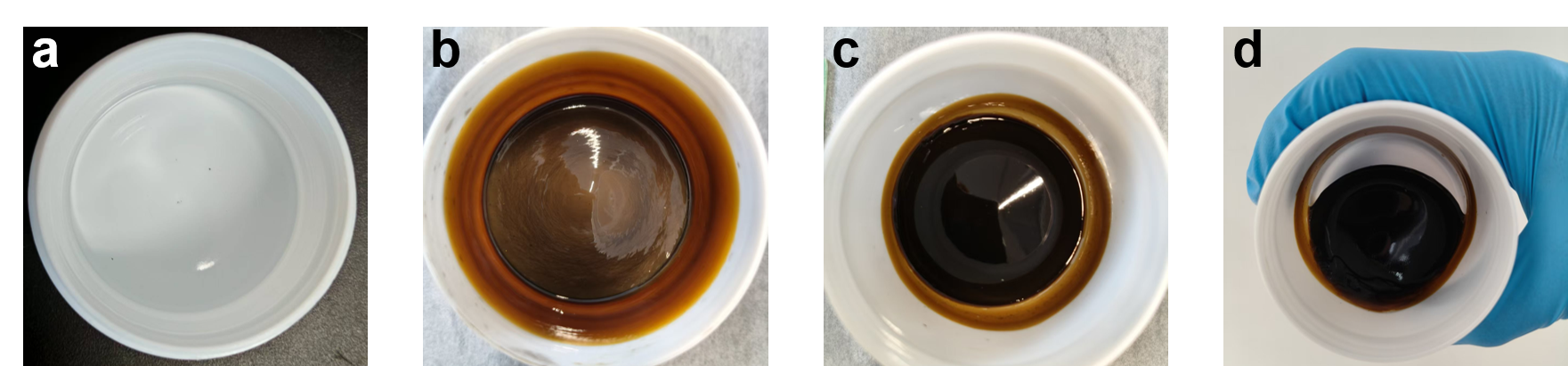


**Fig. S1** **a** image of PAA gel, **b** image of GO (16 mg/ml), **c** mixture of GO aerogel (not being reduced) and PAA by ratio 1:3 (G1P3). **d** Photograph of the colloidal state of the composite ink

**S2** The large-layered structure of GO with disordered stacking **(Fig. S2a, b, g)** and random orientation **(Fig. S2c, d)** affects the dispersibility of two-dimensional sheets in the ink, The nanosheet structure with uneven area size is also not conducive to DIW **(Fig. S2e, f)**, which is not suitable for direct DIW 3D printing.


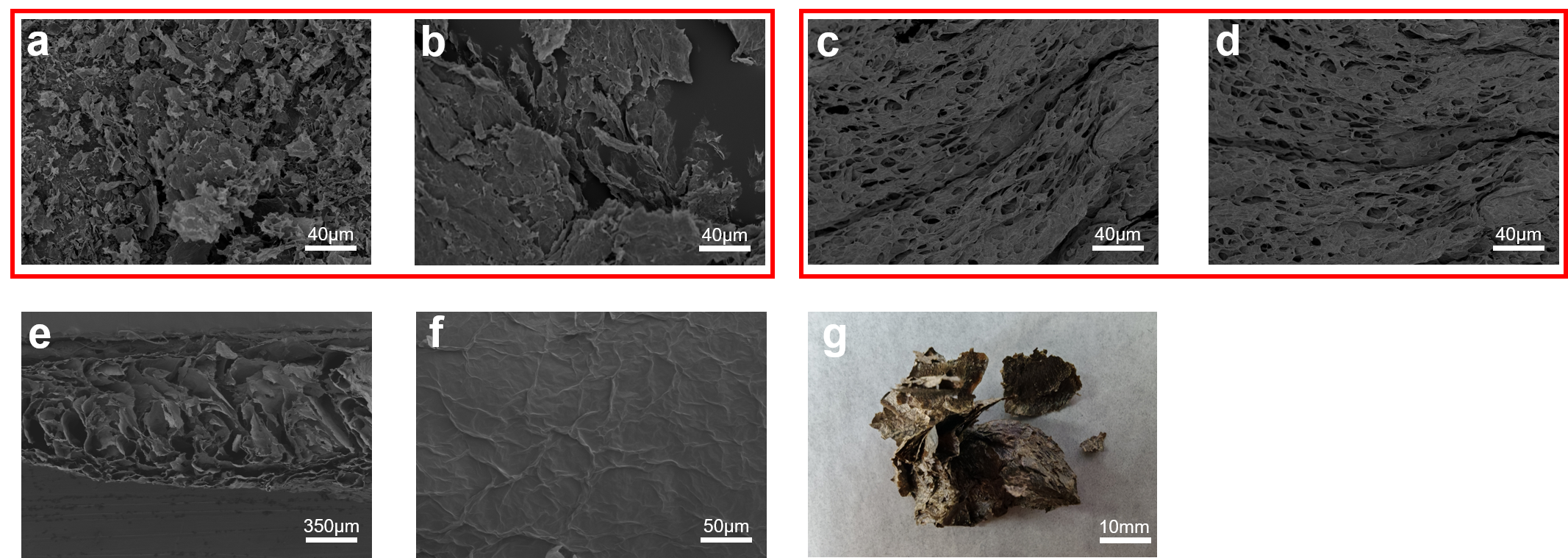


**Fig. S2 a-f** SEM image of GO, **g** is the Optical photo of GO aerogel

**S3** Several pictures showing the states of composite aerogels after freeze-drying are presented. It can be observed that as the content of PAA increases, due to the overly loose framework of the aerogels and the too low content of 2D graphene sheets, it becomes difficult to support the entire structure, and phenomena such as cracking and shrinkage occur in the aerogels.


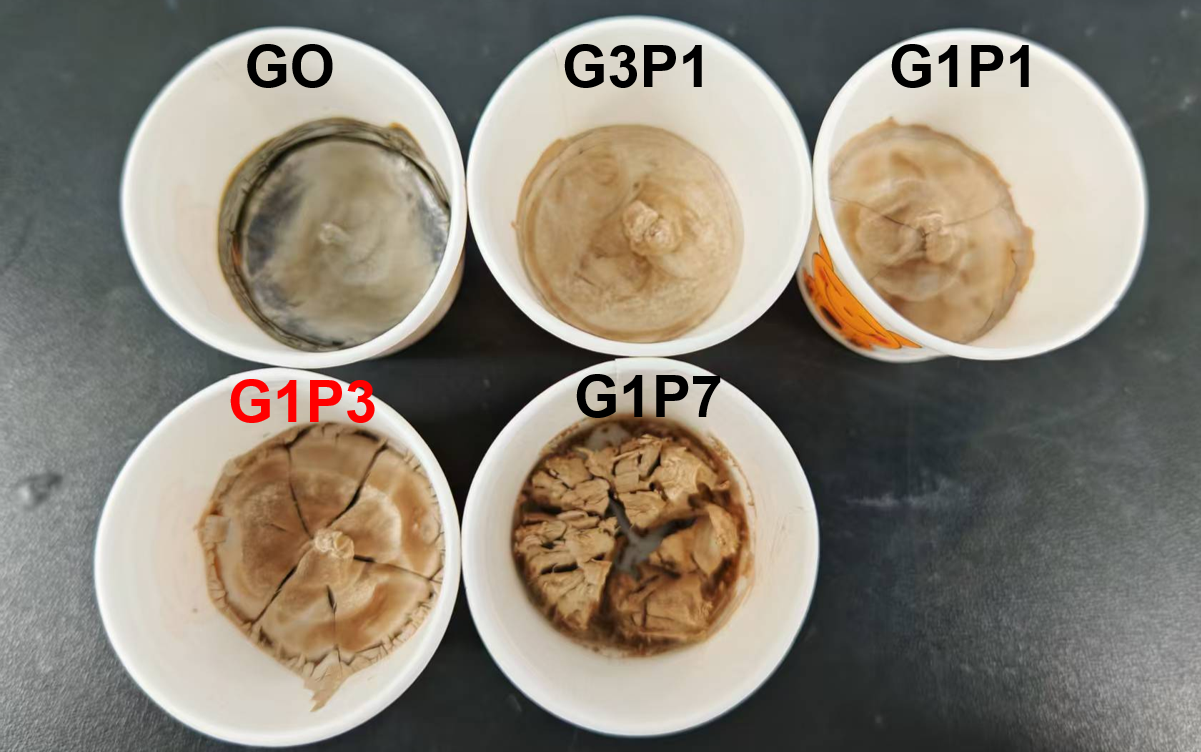


**Fig. S3** image of GO, G3P1, G1P1, G1P3 and G1P7 aerogel

**S4** The thermogravimetric profile in an air environment was measured to demonstrate the thermal stability of rGO/PAA aerogel.

**
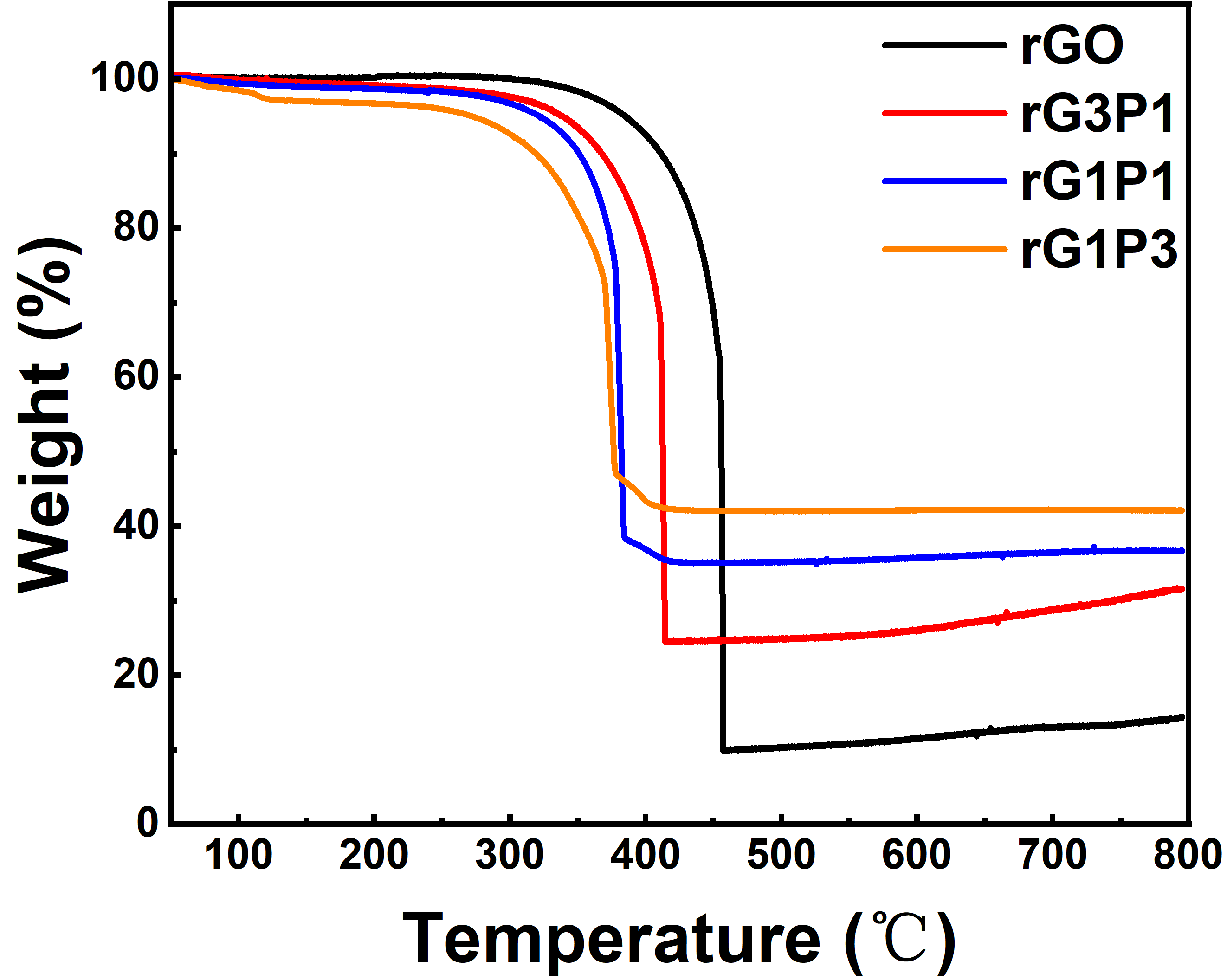
**

**Fig. S4** TGA of rGO/PAA aerogels under Air Atmosphere

**S5** Presentation of the printing performance of G1P3 ink. **Fig. S5a-b** demonstrate the seamless DIW printing of the G1P3 ink, showcasing the high precision of this 3D printing technology. Specifically, **Fig. S5b** (sample under light projection) further highlights the excellent uniformity of the printed structure. **Fig. S5c** displays the framework printed using this high-performance ink. **Fig. S5d** presents the DIW-printed PAA gel framework, exhibiting high fidelity. **Fig. S5e** shows the G1P3 multi-layer framework after liquid nitrogen freezing, maintaining structural integrity under cryogenic conditions. **Fig. S5f** depicts the rG1P3 aerogel framework after thermal reduction. **Fig. S5g** illustrates the framework printed using a coarse needle (inner diameter: 1200 μm). All printed results **(Fig. S5c-g)** exhibit good self-supporting capability. **Fig. S5h-i** compare the printed state and the reduced state of the metamaterial framework (size: 100mm × 40mm × 10mm). Even at this large scale, the structure maintains uniformity, indicating the ink's potential for scalable fabrication. The inner diameters of the printing needles used for subfigures **a-c, d-f, g, and h-i** were 200 μm, 300 μm, 1200 μm, and 1000 μm, respectively. **Fig. S5j** shows the size of the G1P3 aerogel framework before and after reduction, with almost no noticeable volume shrinkage visible to the naked eye.


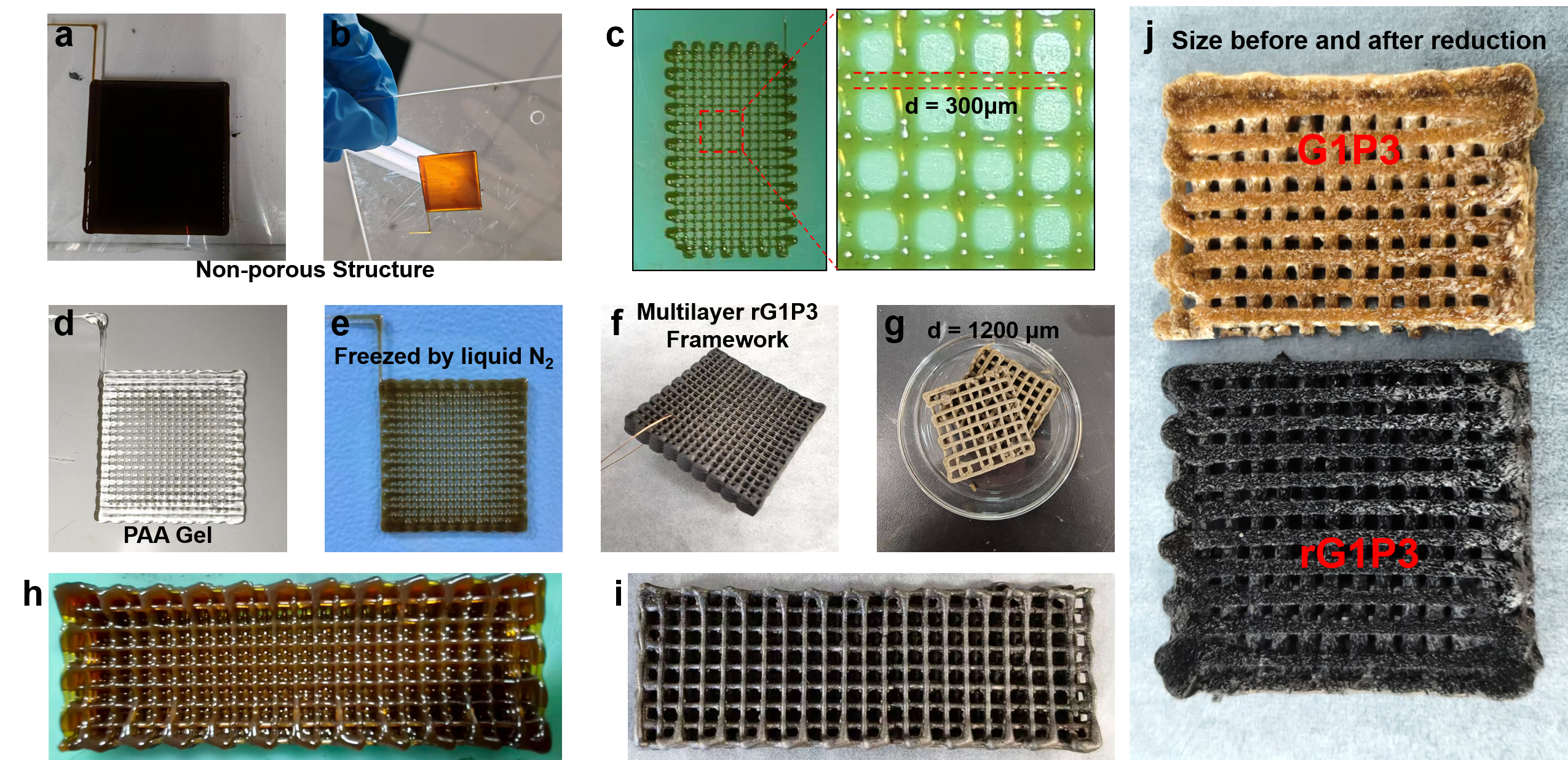


**Fig. S5 a-c** DIW of G1P3 ink. **d** DIW of PAA gel, **e** freezed G1P3 framework by liquid N_2_. **f** multi-layer rG1P3 aerogel framework. **g** DIW 3D printing of framework by large diameter needles (1200 μm). **h** DIW of waveguide sample. **i** thermal reduction of wiveguide sample. **j** Size before and after reduction

**S6** Herein, a printing needle with a diameter of 150 μm was used, and Scanning Electron Microscopy (SEM) observation of the printed framework showed that its line diameter was approximately 200 μm. The ultra-fine printing and structural integrity of the rG1P3 framework are demonstrated. Voids appeared at the connection in the X-Y direction; this is because during the printing transition in the X-Y direction, the height was increased, and due to the effect of gravity, the upper layer lines were slightly deposited into the macroscopic voids of the lower layer.


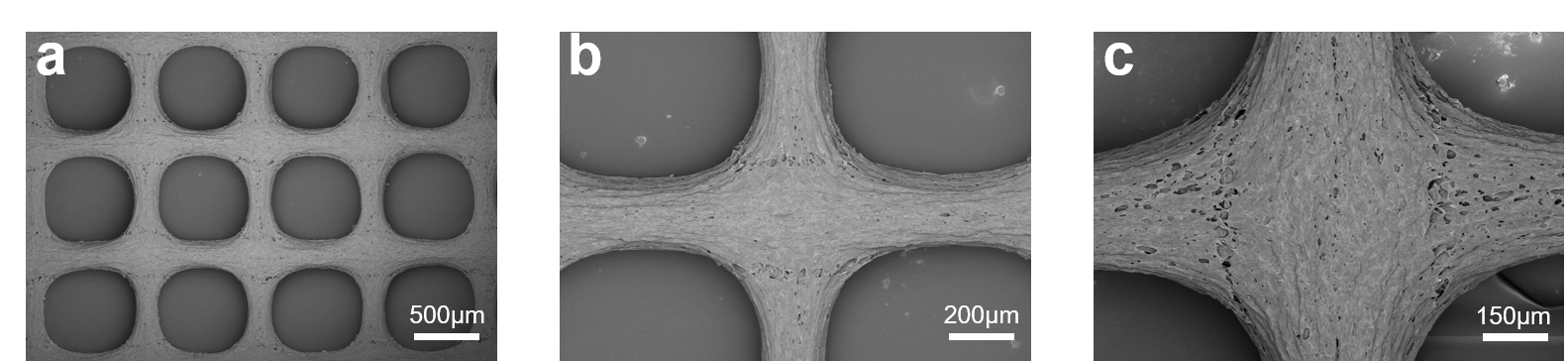
**Fig. S6 a-c** SEM of rG1P3 framework

**S7** Additional extrusion pressure data for different nozzle sizes are provided in **Fig. S7**. The results indicate that when the nozzle diameter is below 600 μm, the axial extrusion force along the printing direction dominates the alignment of graphene layers, causing them to yield and stack preferentially along the extrusion path. In contrast, when the nozzle diameter exceeds 600 μm, the extrusion pressure drops significantly, no obvious morphological changes due to extrusion force can be observed.


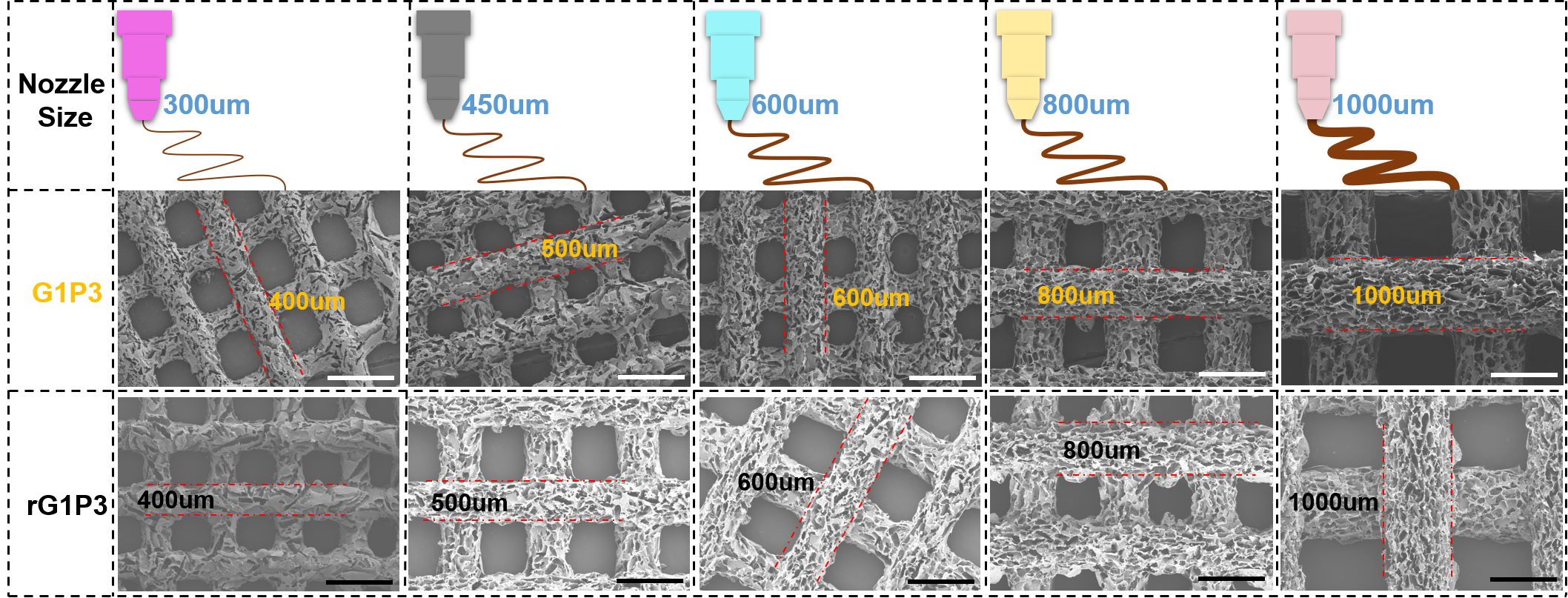
**Fig. S7** SEM images of printed frameworks under different printing needle diameters

**Table S1** Waveguide sample size for different bands

| **Band (GHz)** | **Width (mm)** | **Height (mm)** |
| --- | --- | --- |
| 2.60-3.95 | 72.14 | 34.04 |
| 3.94-5.99 | 47.55 | 22.15 |
| 5.38-8.17 | 34.85 | 15.80 |
| 8.20-12.40 | 22.86 | 10.16 |
| 12.40-18.00 | 15.799 | 7.899 |

**Table S2** Comparison of the present work with those reported in the literature regarding ink

| **Samples** | **Concentration (mg/cm3)** | **Needle size**  **(μm)** | **Printing Resolution (μm)** | **Refs.** |
| --- | --- | --- | --- | --- |
| GO+HPMC | 40 | 300 | 450 | [S1] |
| GO+Sulfur | 50 | 150 | 200 | [S2] |
| GO+BCS | 50 | 300 | 300 | [S3] |
| GO+Ca2+ | 20 | 300 | 300 | [S4] |
| GO + (NH4)2CO3 + SiO2 | 40 | 300 | 300 | [S5] |
| GO + MnO2 +  HPMC | 40 | 300 | 400 | [S6] |
| **This Work (G1P3)** | **4** | **150** | **200** | **/** |

**Table S3** Comparison of the absorption performance of different aerogel-based composite materials

| **Sample** | **RL_min_ (dB)** | **EAB (GHz)** | **Density (mg/cm^3^)** | **Filler Content(%)** | **t_RL_**  **(mm)** | **t_EAB_**  **(mm)** | **Manufact strategy** | **Refs.** |
| --- | --- | --- | --- | --- | --- | --- | --- | --- |
| PI/rGO/CNC-1 | -75.00 | 7.28 | 9.93 | 5 | 3.52 | 2.61 | Mold | [S7] |
| SiC/MWCNTs | -57.36 | 3.34 | 46 | 15 | 2.9 | 1.5 | Mold | [S8] |
| PMN-3 | -64.87 | 6.24 | 9.6 | 1.85 | 1.5 | 1.5 | Mold | [S9] |
| MXene/C-3 | -53.02 | 5.30 | 50 | 4 | 3.8 | 2.4 | Mold | [S10] |
| PI-NC-GAMs | -63.60 | 7.45 | 20 | 1 | 3.43 | 2.57 | Mold | [S11] |
| CSC aerogels | -55.16 | 8.50 | 40 | 1 | 3.4 | 4.5 | Mold | [S12] |
| TP10NSGA800 | -67.64 | 6.12 | 4.08 | 0.45 | 2 | 2.8 | Mold | [S13] |
| 800Co-rGO-pvp | -40.57 | 7.12 | 4.2 | 4 | 2.0 | 2.5 | Mold | [S14] |
| Fe_3_O_4_@C/rGO | -46.20 | 7.92 | 6.2 | 0.7 | 2.8 | 2.8 | Mold | [S15] |
| MG aerogel | -56.85 | 8.10 | 4.0 | 1 | 2.7 | 3.5 | DIW | [S16] |
| **This Work** | **-39.86** | **8.36** | **6.9** | **0.8** | **2.5** | **3.2** | **DIW** | **/** |

**S8** EDS of rGO and rG1P3 **Fig. S8a, b** as well as the 0D/2D interfacial polarization effect. It illustrates the interfacial effect caused by the introduction of PAA after reduction. The originally smooth graphene sheets firmly adsorbed uniform amorphous carbon particles**(Fig. S8c, d, e)**. The XPS C1s of rGO and rG1P3 **(Fig. S9a, b)** aerogels showed reduction of oxygen-containing groups. **Fig. S9c** XRD results show that the characteristic peak of rG1P3 has a large offset compared to the unreduced G1P3 aerogel, indicating the successful reduction of rGO. **Fig. S9d** shows a comparison of the electrical conductivity of four aerogels with different compositions. It can be observed that as the PAA content increases, the electrical conductivity shows a decreasing trend.


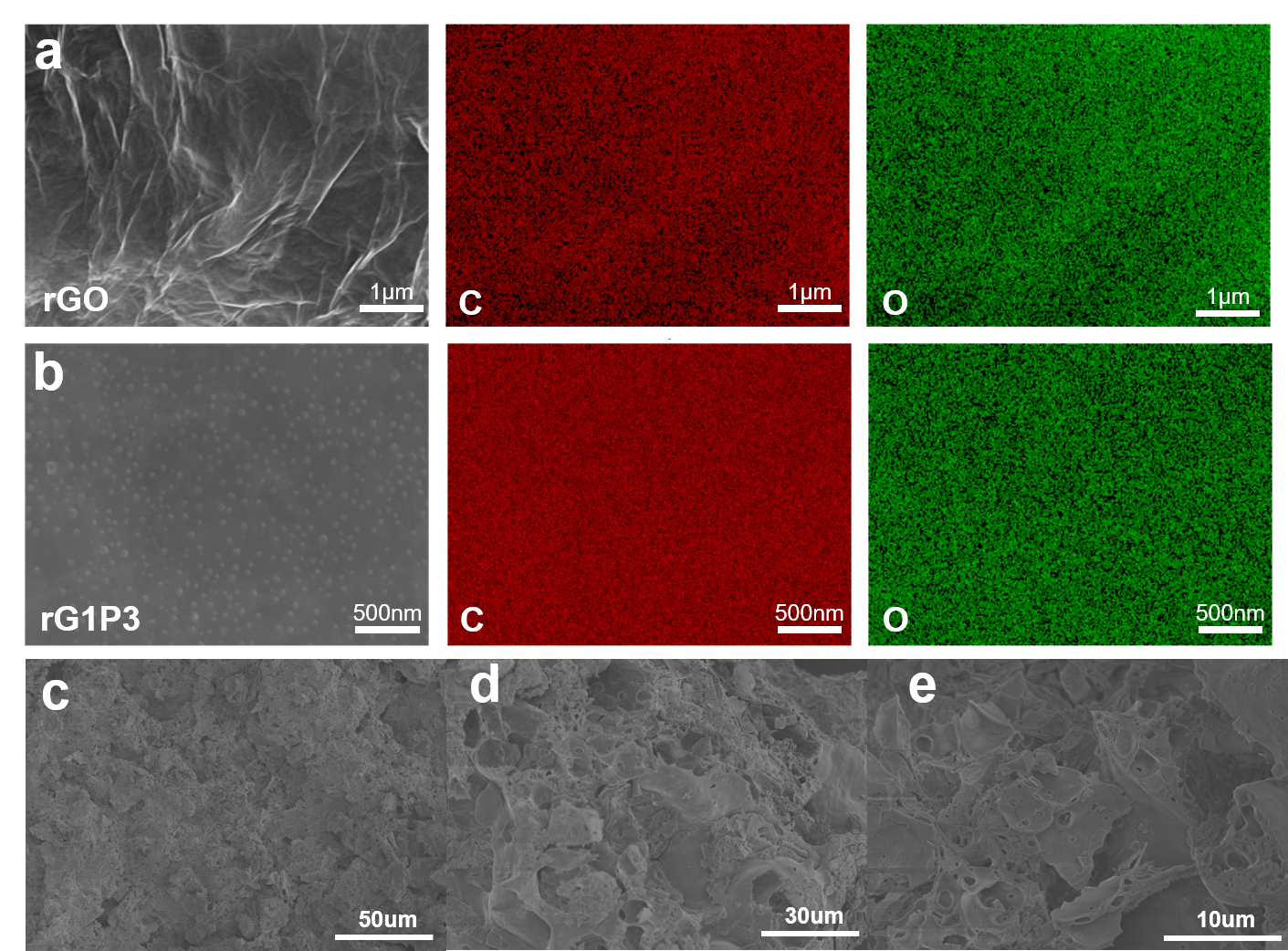


**Fig. S8** SEM and EDS of rGO **a** and rG1P3 **b**, **c-e** SEM of rPAA powder


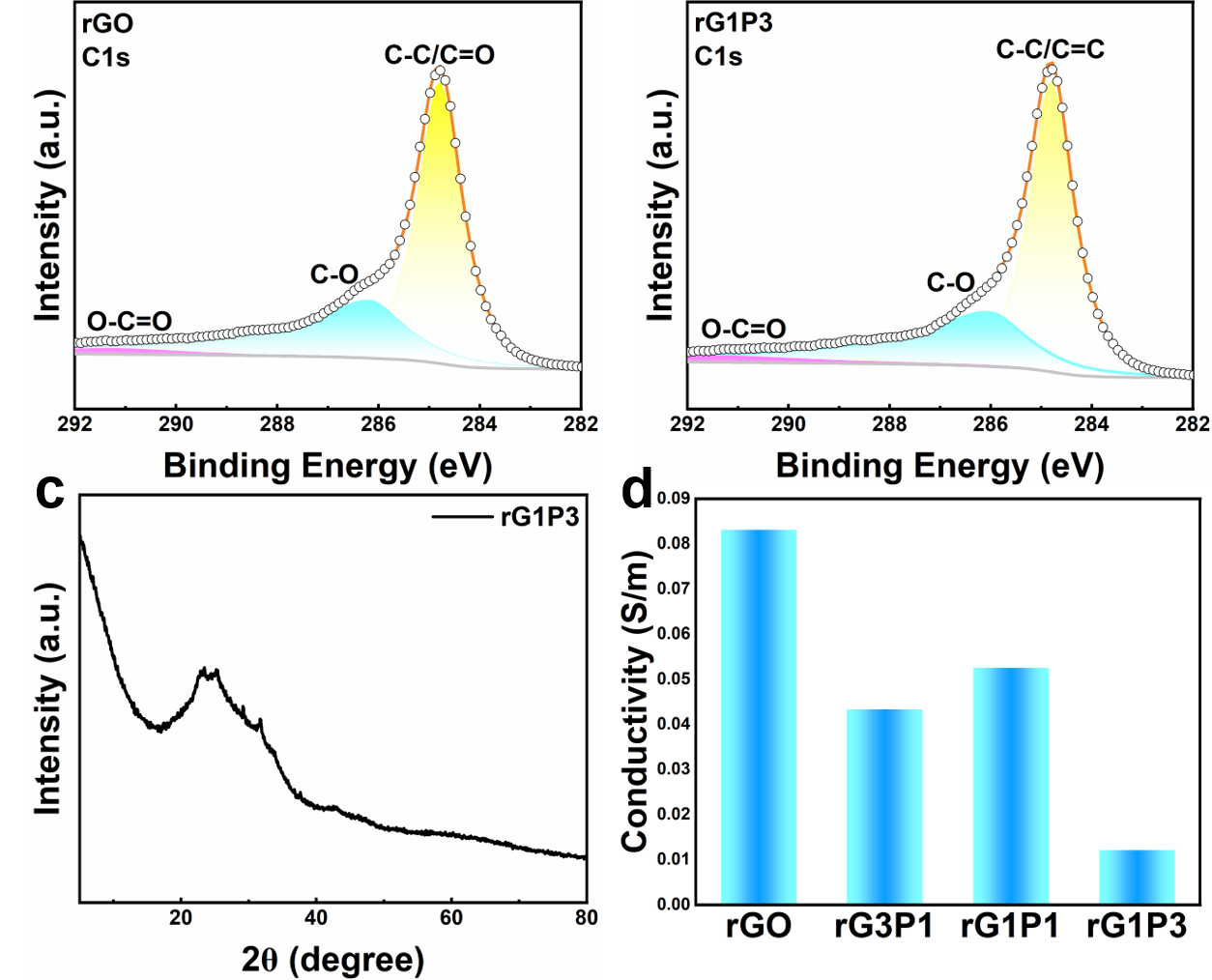


**Fig. S9** XPS C1s of **a** rGO and **b** rG1P3. **c** XRD spectra of rG1P3. **d** Electrical Conductivity of rGO, rG3P1, rG1P1, and rG1P3 Aerogels

**S9** This figure displays the G1P3 aerogel after freeze-drying **(Fig. S10a)**, alongside photographs of G1P3 aerogel and rG1P3 aerogel samples **(Fig. S10b)** before and after thermal annealing in a tube furnace. Fig. S8c shows coaxial samples of the rGxPy (rGO: PAA = x: y) Series Aerogel prepared for electromagnetic measurements. The sample preparation involved vacuum impregnation of bulk rGxPys with molten paraffin wax. After solidification, the samples were precisely cut using a coaxial mold. **(Fig. S10c)** This sample preparation method perfectly preserves the 3D porous structure of the aerogels.


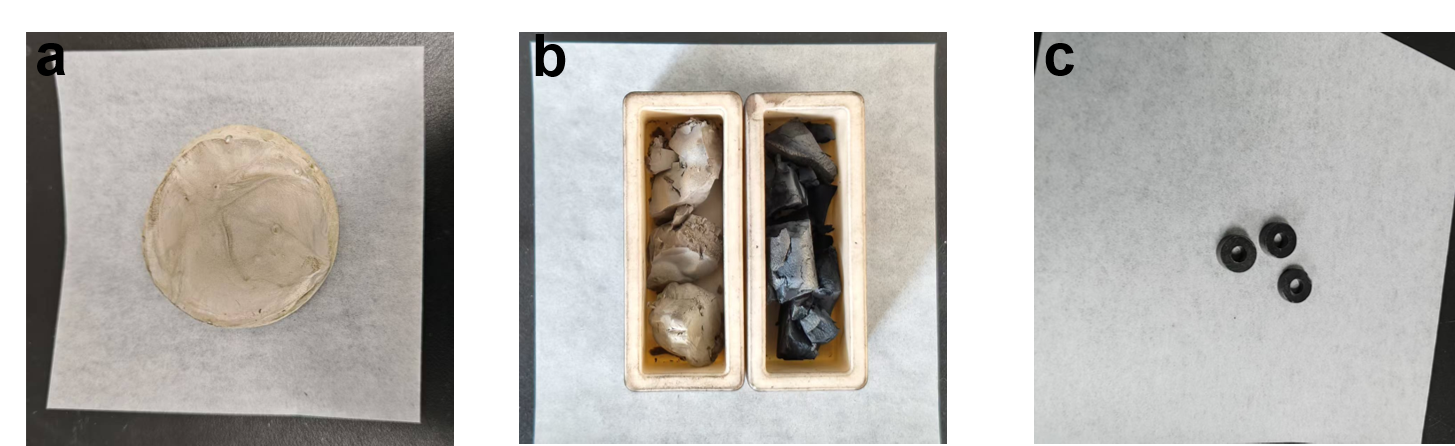


**Fig. S10** **a** Bulk G1P3 aerogel made from a mold. **b** Comparison of aerogel color before and after thermal reduction. **c** Photograph of the coaxial test sample

**S10** Electromagnetic simulation parameter scanning results of material-structure dual-gradient graphene aerogel metamaterial absorbers. **Fig. S11 a-f** depict the variations in **a** the number of superstructure layers, **b-c** material systems, **d** pore size, **e** printed line diameter, and **f** electromagnetic wave incidence angle, respectively. For computational efficiency, we set P1 = 2*P2 in the calculations. **Fig. S12** conducted scanning of the metamaterial's pore size (P2) and diameter (D) with higher sensitivity, based on the parameter scanning results obtained from **Fig. S11**. **Fig. S13** presents a hybrid scanning mode of two structural parameters. **Fig. S14** simulates the additional resonance peaks observed in the measured waveguide data at 9.8 GHz. By reducing the diameter of the printed lines and the aperture of the structure, the thickness of the metamaterial is decreased to match the sample thickness used in the actual waveguide measurements.


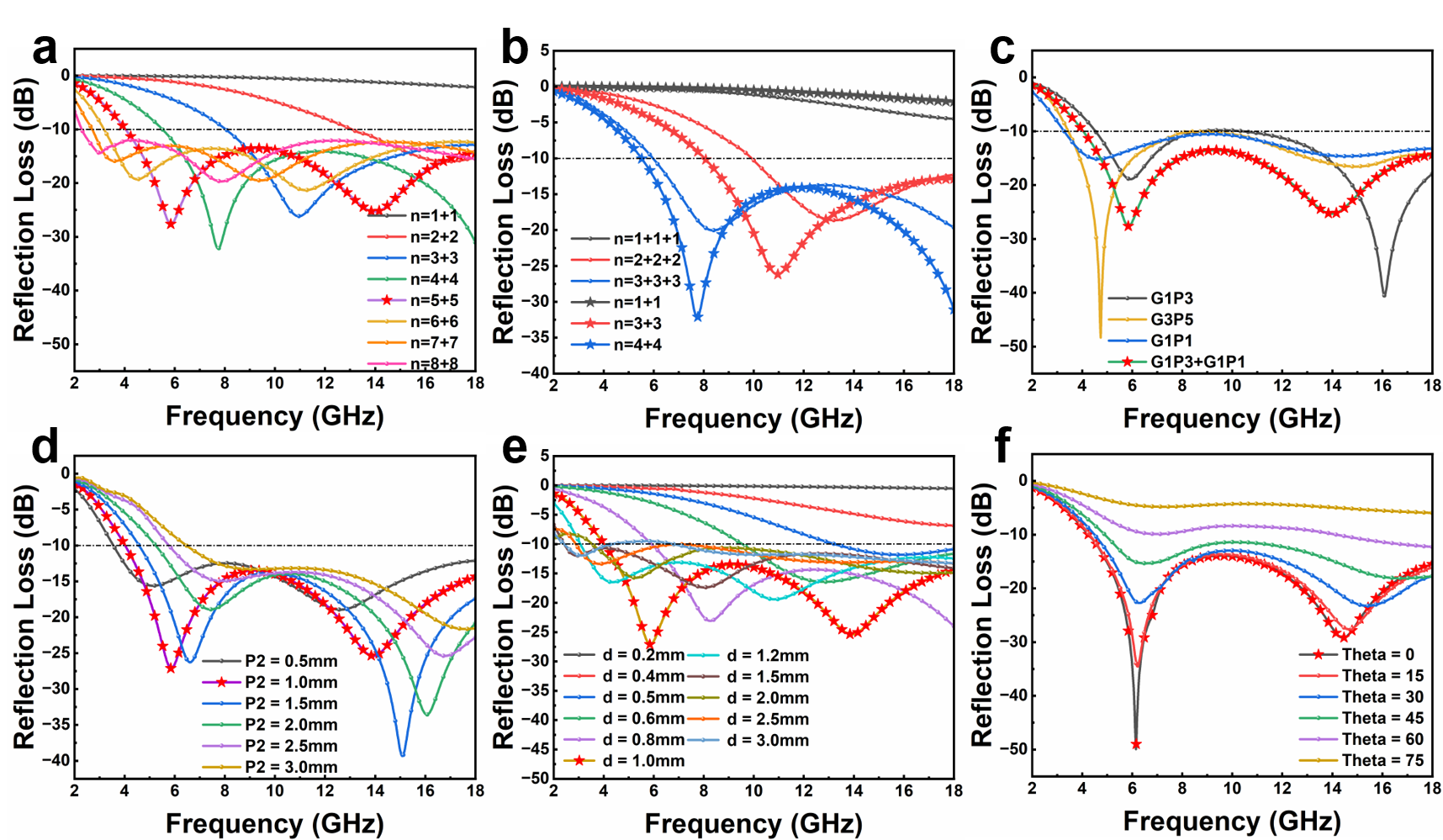


**Fig. S11** Parameter sweeps and optimization of electronmagnetic simulation

**
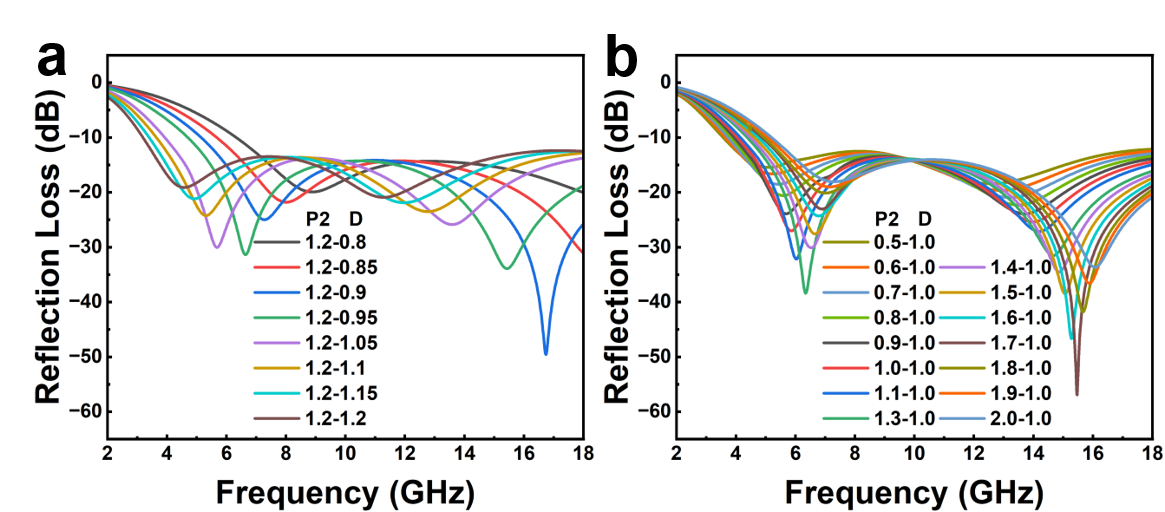
 Fig. S12** Parameter sweeps and optimization of RL simulation


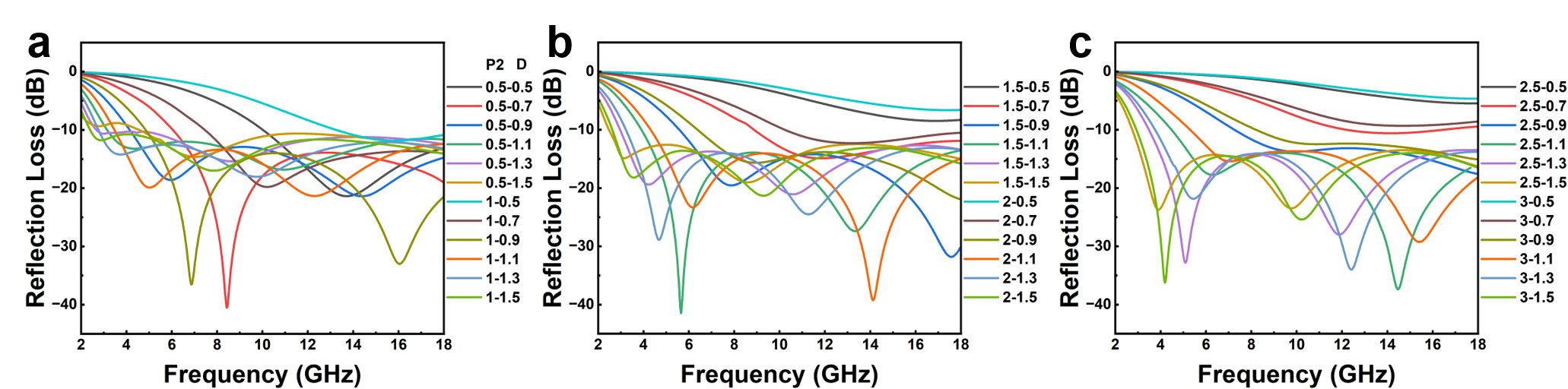


**Fig. S13** Parameter sweeps and optimization of electronmagnetic simulation


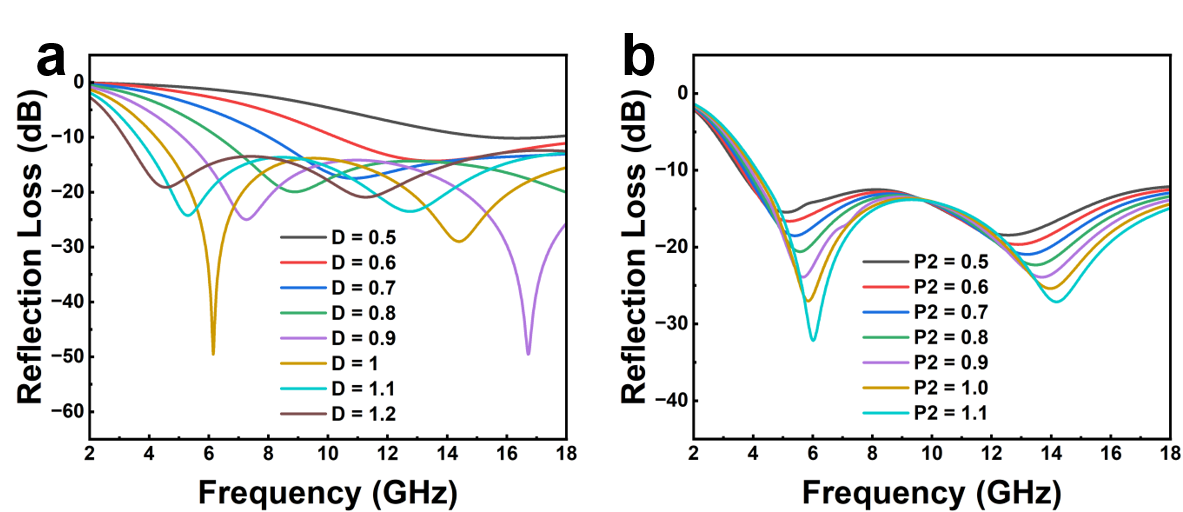
 **Fig. S14** Parameter sweeps and optimization of electronmagnetic simulation

**S11** To further analyze the electromagnetic wave absorption mechanism of the metamaterial absorber at the material-structure level, in addition to simulating the electric field distribution and energy density at the two absorption peak frequencies, we also selected three additional frequencies for simulation, as shown in **Fig. S15**, to supplement the analysis of the electromagnetic absorption mechanism.


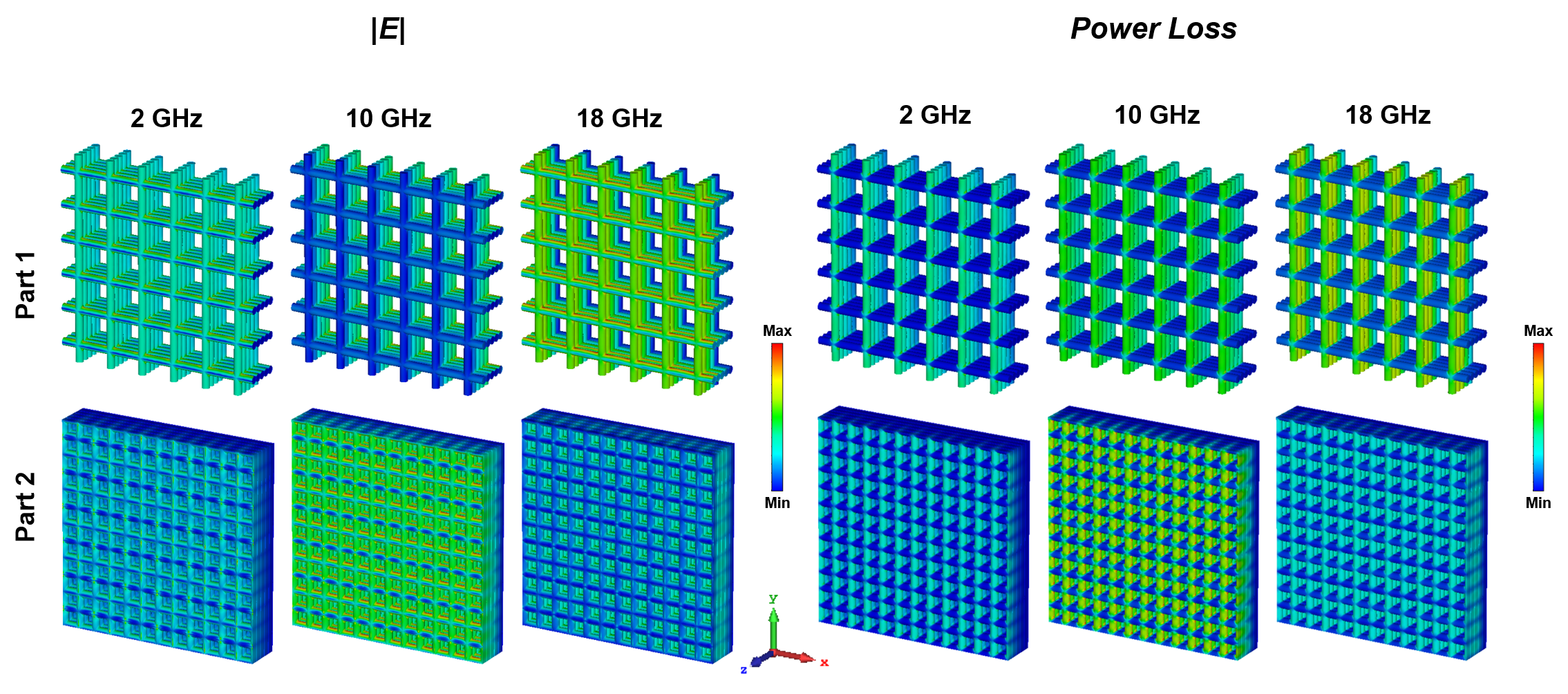


**Fig. S15** Simulation diagrams of electric field distribution and energy loss density of the metamaterial absorber at 2, 10, and 18 GHz

**Supplementary References**

1. B. Yao, S. Chandrasekaran, H. Zhang, A. Ma, J. Kang et al., 3D-printed structure boosts the kinetics and intrinsic capacitance of pseudocapacitive graphene aerogels. Adv. Mater. **32**(8), e1906652 (2020). <https://doi.org/10.1002/adma.201906652>
2. K. Shen, H. Mei, B. Li, J. Ding, S. Yang, 3D printing sulfur copolymer-graphene architectures for Li-S batteries. Adv. Energy Mater. **8**(4), 1701527 (2018). <https://doi.org/10.1002/aenm.201701527>
3. E. García-Tuñon, S. Barg, J. Franco, R. Bell, S. Eslava et al., Printing in three dimensions with graphene. Adv. Mater. **27**(10), 1688–1693 (2015). <https://doi.org/10.1002/adma.201405046>
4. Y. Jiang, Z. Xu, T. Huang, Y. Liu, F. Guo et al., Direct 3D printing of ultralight graphene oxide aerogel microlattices. Adv. Funct. Mater. **28**(16), 1707024 (2018). <https://doi.org/10.1002/adfm.201707024>
5. C. Zhu, T.Y. Han, E.B. Duoss, A.M. Golobic, J.D. Kuntz et al., Highly compressible 3D periodic graphene aerogel microlattices. Nat. Commun. **6**, 6962 (2015). <https://doi.org/10.1038/ncomms7962>
6. B. Yao, S. Chandrasekaran, J. Zhang, W. Xiao, F. Qian et al., Efficient 3D printed pseudocapacitive electrodes with ultrahigh MnO_2_ loading. Joule **3**(2), 459–470 (2019). <https://doi.org/10.1016/j.joule.2018.09.020>
7. S. Shao, S. Xing, K. Bi, T. Zhao, H. Wang et al., Fabrication of graphene/polyimide/Co-N-C aerogel with reinforced electromagnetic losses and broadband absorption for highly efficient microwave absorption and thermal insulation. Chem. Eng. J. **494**, 152976 (2024). <https://doi.org/10.1016/j.cej.2024.152976>
8. Y. Xia, Z. Zhang, K. Li, S. Zhao, G. Chen et al., Lightweight and high-strength SiC/MWCNTs nanofibrous aerogel derived from RGO/MWCNTs aerogel for microwave absorption. Chem. Eng. J. **486**, 150417 (2024). <https://doi.org/10.1016/j.cej.2024.150417>
9. Y. Wang, Z. Qu, W. Wang, H. Qian, X. Song et al., Multidimensional nanomaterials synergistic polyimide nanofiber/MXene/NiFe_2_O_4_ hybrid aerogel for high-performance microwave absorption. Chem. Eng. J. **470**, 144435 (2023). <https://doi.org/10.1016/j.cej.2023.144435>
10. F. Wu, P. Hu, F. Hu, Z. Tian, J. Tang et al., Multifunctional MXene/C aerogels for enhanced microwave absorption and thermal insulation. Nano-Micro Lett. **15**(1), 194 (2023). <https://doi.org/10.1007/s40820-023-01158-7>
11. Y. Cai, Z. Wang, G. Fei, M. Lavorgna, H. Xia, Polyimide derived carbon/graphene hybrid aerogel microspheres for strong and wide bandwidth microwave absorption. Adv. Funct. Mater. **35**(25), 2419252 (2025). <https://doi.org/10.1002/adfm.202419252>
12. X. Wang, Y. Yuan, X. Sun, R. Qiang, Y. Xu et al., Lightweight, flexible, and thermal insulating carbon/SiO(2)@CNTs composite aerogel for high-efficiency microwave absorption. Small **20**(30), e2311657 (2024). <https://doi.org/10.1002/smll.202311657>
13. X. Huang, L. Zhang, G. Yu, J. Wei, G. Shao, Polarization genes dominated heteroatom-doped graphene aerogels toward super-efficiency microwave absorption. J. Mater. Chem. C **11**(29), 9804–9814 (2023). <https://doi.org/10.1039/d3tc01965a>
14. K. Cao, X. Yang, Y. Zhang, J. Wen, J. Chen et al., Preparation of magnetic three-dimensional porous Co-rGO aerogel for enhanced microwave absorption. Carbon **208**, 111–122 (2023). <https://doi.org/10.1016/j.carbon.2023.03.037>
15. X. Huang, J. Wei, Y. Zhang, B. Qian, Q. Jia et al., Ultralight magnetic and dielectric aerogels achieved by metal-organic framework initiated gelation of graphene oxide for enhanced microwave absorption. Nano-Micro Lett. **14**(1), 107 (2022). <https://doi.org/10.1007/s40820-022-00851-3>
16. X. Liu, B. Zheng, Y. Hua, S. Lu, Z. Nong et al., Ultralight MXene/rGO aerogel frames with component and structure controlled electromagnetic wave absorption by direct ink writing. Carbon **230**, 119650 (2024). <https://doi.org/10.1016/j.carbon.2024.119650>
