# Supplementary figures and images for: Multiscale Design of Dual-Gradient Metamaterials Using Gel-Mediated 3D-Printed Graphene Aerogels for Broadband Electromagnetic Absorption
Source: Nanomicro Lett. 2026 Jan 5;18:162. doi: 10.1007/s40820-025-02005-7 (PMC12765778; doi:10.1007/s40820-025-02005-7)

## Slide 1
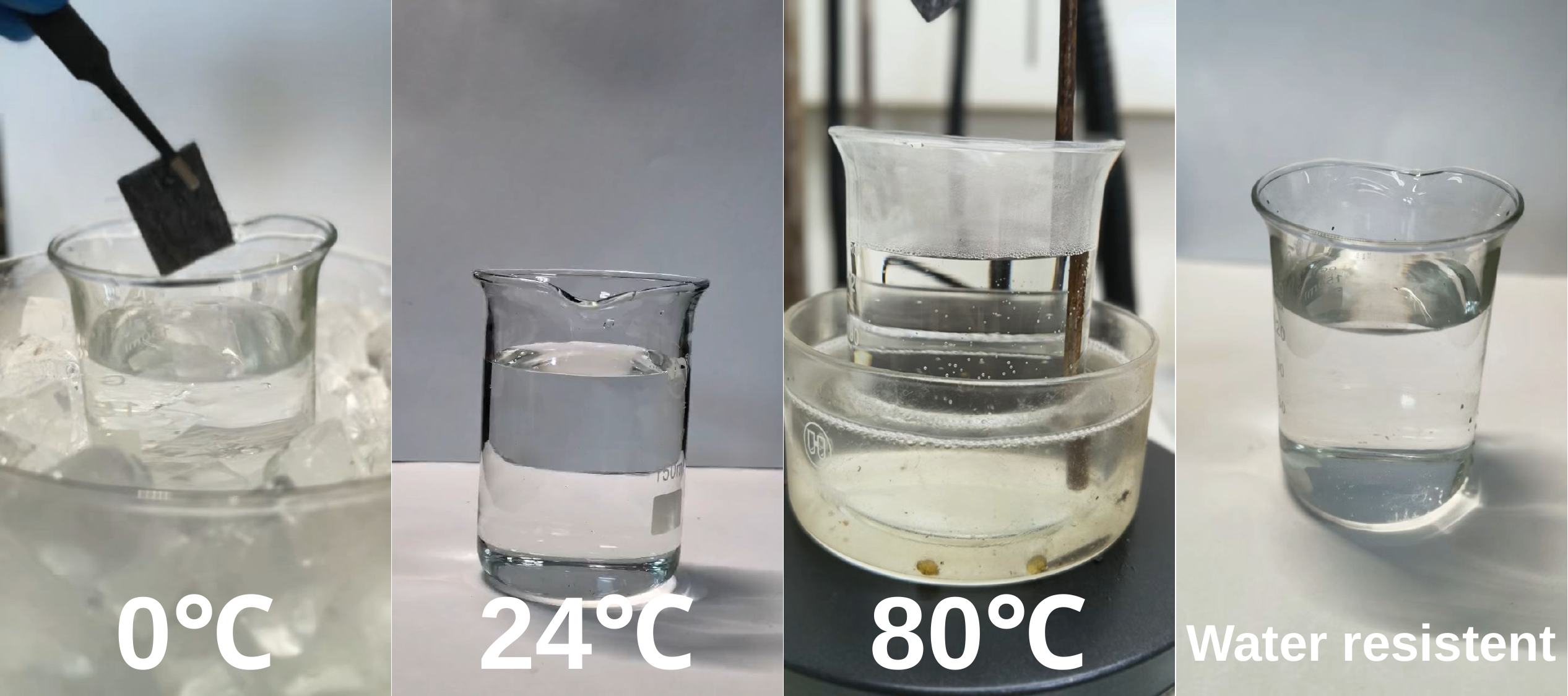

24℃
80℃
0℃
Water resistent

Supplement: Supplementary file 2 — Supplementary file2 (PPTX 11859 kb) [file 40820_2025_2005_MOESM2_ESM.pptx]
